# A bibliometric review of deep learning in crop monitoring: trends, challenges, and future perspectives

**DOI:** 10.3389/frai.2025.1636898

**Published:** 2025-09-17

**Authors:** Rui Zhang, Xue Wu, Jing Li, Pengyu Zhao, Qing Zhang, Lige Wuri, Donghui Zhang, Zhijie Zhang, Linnan Yang

**Affiliations:** ^1^The Key Laboratory for Crop Production and Smart Agriculture of Yunnan Province, Kunming, China; ^2^Yunnan Agricultural University, Kunming, China; ^3^Kunming Institute of Eco-Environmental Sciences, Kunming, China; ^4^Shanxi Key Laboratory of Earth Surface Processes and Resource Ecology Security in Fenhe River Basin, Taiyuan Normal University, Jinzhong, China; ^5^Xinjiang Center for Ecological Meteorology and Satellite Remote Sensing, Urumqi, China; ^6^School of Government, Beijing Normal University, Beijing, China; ^7^Faculty of History and Tourism Culture, Inner Mongolia Minzu University, Tongliao, China; ^8^Institute of Remote Sensing Satellite, China Academy of Space Technology, Beijing, China; ^9^School of Geography, Development and Environment, The University of Arizona, Tucson, AZ, United States

**Keywords:** deep learning, crop monitoring, machine learning, precision agriculture, VOSviewer, CiteSpace, bibliometric analysis, knowledge graph

## Abstract

Global agricultural systems face unprecedented challenges from climate change, resource scarcity, and rising food demand, requiring transformative solutions. Artificial intelligence (AI), particularly deep learning (DL), has emerged as a critical tool for agricultural monitoring, yet a systematic synthesis of its applications remains understudied. This paper presents a comprehensive bibliometric and knowledge graph analysis of 650 + publications (2000–2024) to map AI’s role in agricultural information identification, with emphasis on DL and remote sensing integration (e.g., UAVs, satellites). Results highlight Convolutional Neural Networks (CNNs) as the dominant technology for real-time crop monitoring but reveal three persistent barriers: (1) scarcity of annotated datasets, (2) poor model generalization across environments, and (3) challenges in fusing multi-source data. Crucially, interdisciplinary collaboration—though vital for scalability—is identified as an underdeveloped research frontier. It is concluded that while AI can revolutionize agriculture, its potential hinges on improving data quality, developing environment-adaptive models, and fostering cross-domain partnerships. This study provides a strategic framework to accelerate AI’s integration into global agricultural systems, addressing both technical gaps and policy needs for future food security.

## 1 Introduction

Agriculture, a fundamental pillar of human civilization, now faces a perfect storm of challenges driven by a rapidly expanding global population, climate change, dwindling natural resources, and an urgent need for more sustainable production systems (Ahmad et al., 2021; Abbass et al., 2022). As the global population is projected to reach nearly 10 billion by 2050, the demand for food will increase exponentially, placing immense pressure on existing agricultural systems (Abdullahi et al., 2015). Yet, conventional agricultural practices, often reliant on manual labor, limited data processing, and slow response times, are increasingly unable to meet the rising demands for faster, more accurate, and scalable solutions (Chen et al., 2021; Chklovski et al., 2023). The urgency of this challenge cannot be overstated: food security, sustainability, and environmental preservation are all at stake (Jackson-Davis et al., 2023). As climate change alters rainfall patterns, intensifies pest outbreaks, and modifies growing seasons, the agricultural sector must embrace technological advancements to navigate this uncertainty and ensure resilient food production systems (Alahi et al., 2023).

In this context, machine learning (ML) and artificial intelligence (AI) emerge as critical tools capable of revolutionizing agricultural practices (Alahi et al., 2023; Chklovski et al., 2023). These technologies offer transformative potential by enabling high-throughput, data-driven decision-making that can drastically improve efficiency, precision, and sustainability in agriculture (Abbasi and Erdebilli, 2023). By leveraging AI-powered systems for tasks such as crop disease detection, pest management, and yield forecasting, farmers can optimize resource usage, minimize environmental impact, and maximize crop productivity (Abbas et al., 2024). These technologies also hold the promise of increasing agricultural resilience by providing early warnings and actionable insights, allowing for rapid intervention in the face of pest infestations or plant diseases (Lu et al., 2017; Tassis et al., 2021). Ultimately, integrating AI into agriculture is not just a matter of technological innovation—it is a vital step towards ensuring global food security in the face of mounting environmental pressures. The research addressed in this paper explores the application of deep learning and remote sensing technologies to address these critical needs, offering a comprehensive approach to agricultural information identification and monitoring (Lu et al., 2017).

### 1.1 Research landscape

The intersection of machine learning and agriculture has witnessed tremendous growth over the past few decades, evolving from rudimentary applications of traditional machine learning models to the sophisticated deep learning architectures employed today (Abbas I. et al., 2021). Early research in agricultural data analysis largely focused on conventional machine learning methods, including decision trees (Abdar et al., 2021b; Zhang et al., 2025b), support vector machines (Pérez et al., 2017), and k-nearest neighbors (Zhang et al., 2020), applied to relatively simple datasets such as crop disease classification or pest detection. While these models proved useful in certain contexts, their limitations in handling large, complex datasets became apparent as agriculture entered the era of big data.

With the advent of deep learning in the 2010s, a paradigm shift occurred in agricultural research. Convolutional Neural Networks (CNNs) and other deep learning models revolutionized the way crop monitoring tasks, such as disease identification and pest detection, were performed (Bao et al., 2024). These models excel at recognizing complex patterns in vast datasets, such as high-resolution images captured by drones and satellites, which traditional machine learning algorithms could not process as effectively (Abdulkadirov et al., 2023). The combination of deep learning with remote sensing technologies has been particularly impactful, allowing for high-resolution, real-time monitoring of crops over large areas (Abbas I. et al., 2021). UAVs, satellites, and ground-based sensors have enabled researchers and farmers alike to gather massive amounts of data, which can be processed to extract meaningful insights that were previously impossible to obtain (Jumaat et al., 2018).

The evolution of this research has been global in nature, with countries such as China, India, the United States, Japan, and various European nations, including the United Kingdom, Germany, and France, leading the charge in advancing AI-powered agricultural technologies (Alola et al., 2023; Dimyati et al., 2023; European Assoc Study Live et al., 2023; Guo et al., 2023; Abbas et al., 2024; Adger et al., 2024). These nations have invested heavily in AI-driven agricultural technologies, with numerous advancements made in crop health monitoring, pest management, and the optimization of resource use (Loeffler et al., 2024). In China, for instance, AI-powered systems have been employed for real-time pest surveillance, while India has seen significant progress in the use of deep learning for disease diagnosis in crops like rice and tomatoes (Xue et al., 2024). As AI techniques continue to mature, the scope of applications has expanded from image classification and disease detection to more sophisticated systems that can predict crop yields, monitor soil health, and even automate tasks like harvesting (Tassis et al., 2021). While the research is progressing rapidly, there remains much to be done to overcome challenges in scalability, data heterogeneity, and system integration, which are crucial for ensuring that these innovations can be widely adopted by farmers globally.

### 1.2 Research challenges

Despite the remarkable progress made in integrating machine learning and remote sensing technologies into agriculture, several significant challenges must be addressed for these technologies to reach their full potential:

- High-Quality, Labeled Datasets: One primary challenge is the need for high-quality, labeled datasets. Accurate classification of images, pest detection, or disease diagnosis by deep learning models requires vast amounts of data (Chou et al., 2019). However, acquiring such datasets is resource-intensive, requiring significant manual labor for data collection and annotation (Appels et al., 2018; Bojar et al., 2022).
- Model Generalization Across Regions: Agricultural data is inherently heterogeneous, varying significantly across crops, geographic locations, and environmental conditions. Models trained on datasets from one region may fail to generalize to others due to differences in climate, soil types, or farming practices (Guo et al., 2023; Rubbens et al., 2023). This challenge requires further research into improving the adaptability of models across diverse agricultural environments.
- Deployment in Real-World Agricultural Environments: AI models may perform exceptionally in controlled settings but degrade when faced with the dynamic and unpredictable conditions of agricultural fields. Variations in lighting, occlusions caused by plant leaves, and environmental noise can affect model accuracy (Gehlot et al., 2023). Ensuring effective deployment across diverse environments is essential for practical use (Abedi et al., 2025).
- Multi-Source Data Integration: The integration of multi-source data—such as satellite imagery, UAV-captured photos, and on-the-ground sensor data—remains a complex issue (Wang et al., 2023). Combining these data sources requires sophisticated algorithms capable of processing large, multi-dimensional datasets in real-time, often demanding significant computational resources (Guo et al., 2024).

### 1.3 Approach and methodology

This study seeks to address these challenges by employing a rigorous bibliometric analysis and knowledge graph methodology to map the evolution of research in agricultural information identification and monitoring (Chaw and Mokji, 2017). By systematically analyzing the body of literature on the application of machine learning and remote sensing technologies in agriculture, this paper identifies key trends, breakthroughs, and gaps in current research. The knowledge graph approach offers a visual representation of the interconnectedness of various research topics, providing insights into how machine learning and AI have been integrated into agricultural practices over time (Abubakar et al., 2022; Ahmed et al., 2023; Al-Shammary et al., 2024).

In addition to the bibliometric analysis, this paper presents a detailed examination of the technical aspects involved in the integration of AI and remote sensing technologies. Specifically, it explores how deep learning models, such as Convolutional Neural Networks (CNNs) and Transfer Learning, are being used to analyze crop images, detect diseases, and identify pests (Chen et al., 2020). Furthermore, the paper delves into the challenges associated with the use of remote sensing technologies, such as UAVs and satellite imagery, in large-scale agricultural monitoring (Zhang et al., 2025a). By synthesizing existing literature and examining case studies from different regions and crop types, this research provides a comprehensive overview of the current state of AI-driven agricultural monitoring systems and outlines the future directions necessary to overcome the existing barriers.

The motivation for this study stems from the increasing challenges faced by global agricultural systems, including climate change, resource scarcity, and the growing demand for food. Traditional farming methods are becoming insufficient to meet these demands, necessitating the integration of advanced technologies for sustainable, efficient agricultural practices. Machine learning, particularly deep learning, has demonstrated its potential to revolutionize agricultural monitoring by improving accuracy in crop disease detection, pest management, and yield forecasting. However, significant gaps remain, particularly in data quality, model generalization, and multi-source data integration (Seiya et al., 2020; Wei et al., 2025). This study aims to address these gaps by developing AI models that can adapt to diverse agricultural environments, improve crop monitoring accuracy, and promote sustainable farming practices, ultimately contributing to global food security.

The current study provides several key contributions to advancing AI-driven agricultural practices, particularly in crop monitoring, pest detection, and disease diagnosis. First, this research addresses the challenges associated with integrating deep learning algorithms and remote sensing technologies, proposing a novel framework that combines these tools for more accurate, real-time crop monitoring. A critical contribution of this study is the development of an advanced deep learning model tailored to agricultural applications, showing significant improvements in pest detection and disease diagnosis compared to traditional methods. Furthermore, this study introduces a model capable of generalizing across diverse agricultural environments, which is essential for adapting to varying crops, pests, and climatic conditions without requiring extensive retraining. Additionally, by improving the precision of agricultural practices, the research contributes to more sustainable farming, optimizing resource use while enhancing crop yield predictions and pest control measures. These contributions lay the groundwork for future AI-driven agricultural systems, offering innovative solutions to some of the most pressing challenges in global food security and agricultural sustainability.

In conclusion, this study not only highlights the advancements in AI and remote sensing for agriculture but also provides a roadmap for future research, pointing out key areas that require further exploration, such as data standardization, model generalization, and multi-source data integration. The manuscript begins with a comprehensive background on the challenges faced by global agricultural systems and the potential of AI in addressing these issues. It then explores the integration of deep learning models with remote sensing technologies for crop monitoring. The subsequent sections focus on the methodology, data analysis, and key findings related to improving model generalization and enhancing agricultural sustainability. Finally, the manuscript concludes by discussing the implications of the study and the future directions for AI-driven agricultural systems. Thank you for your valuable feedback. Advancing the understanding of these technologies and addressing their limitations will unlock their full potential to transform agricultural practices, enhancing sustainability, efficiency, and the capacity to meet the growing demands of global food production.

## 2 Data sources and analysis methods

### 2.1 Retrieval strategy and data collation

**Figure 1:**
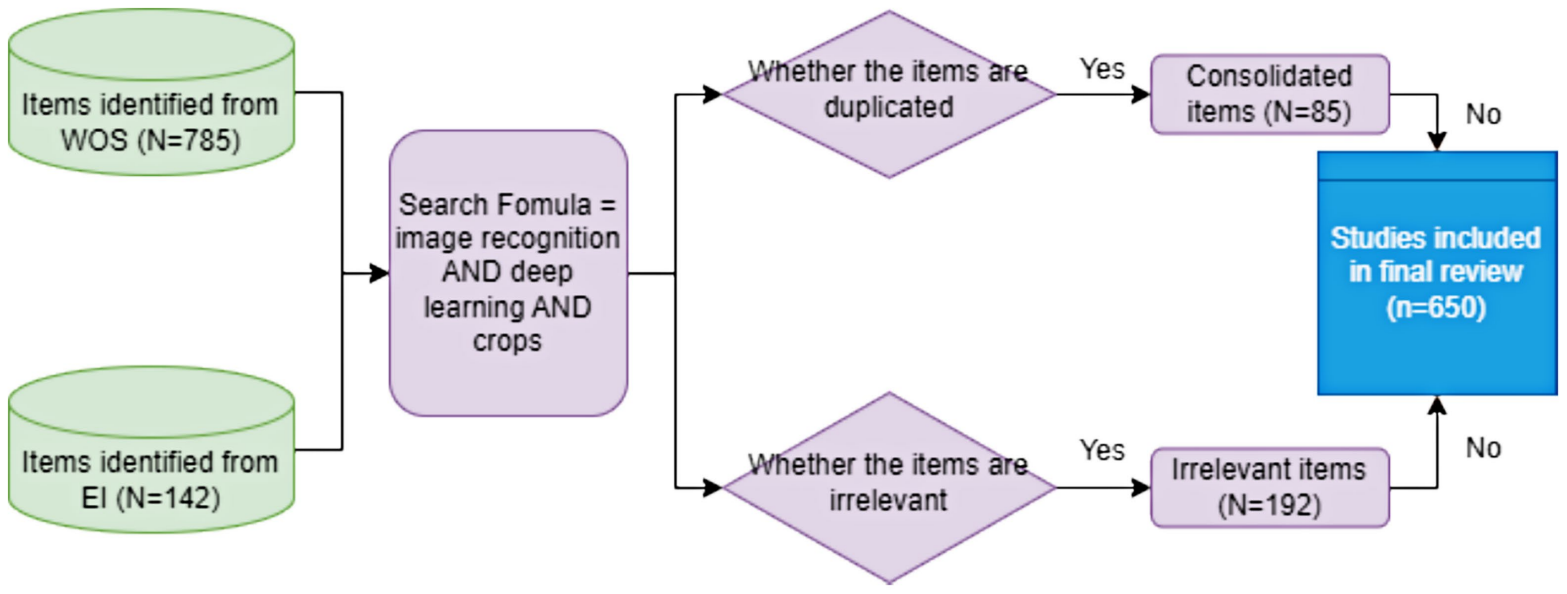
Procedure for screening and selecting eligible studies in the systematic review.

The literature search was conducted using the Web of Science (WOS) and Engineering Index (EI) databases, two of the most authoritative academic databases for multidisciplinary research (Ma et al., 2023). WOS, developed by Clarivate (formerly Thomson Reuters), encompasses the Science Citation Index (SCI), Social Sciences Citation Index (SSCI), and Arts & Humanities Citation Index (A&HCI), renowned for its rigorous journal selection and citation indexing system that enables comprehensive literature retrieval through multiple search parameters including keywords, authors, and DOI. Similarly, EI, established in 1884 by the American Federation of Engineers, serves as a foundational database in engineering disciplines with global recognition. The search strategy employed the query TS = (“image recognition” AND “deep learning” AND “crops”) within the WOS Core Collection, covering all editions and publication years (2000–2025), which initially retrieved 785 records. A corresponding search in EI up to the present date yielded 142 records. After cross-database comparison and removal of 192 irrelevant publications and 85 consolidated items, 650 valid records were retained for analysis, including five highly representative studies that exemplify key advancements in deep learning-based image recognition for crop analysis (Figure 1). This systematic approach ensured a rigorous and comprehensive collection of relevant literature in the field.

In recent years, the application of deep learning technologies in agriculture, particularly for crop disease and pest detection, has seen significant advancements. With the modernization of agricultural production, traditional manual monitoring methods are no longer sufficient to meet the demand for fast and accurate detection. As a result, using deep learning models for disease and pest identification has become an essential tool for improving agricultural productivity. One study focuses on the automatic identification and diagnosis of rice diseases using deep convolutional neural networks (CNNs), providing a powerful framework for disease detection that enables early intervention to ensure crop health (Lu et al., 2017). Another research explores the use of deep learning, particularly Mask-RCNN, for strawberry harvesting robots, contributing to agricultural robotics by optimizing fruit detection, which increases efficiency and reduces labor costs (Yu et al., 2019). A third paper examines the use of deep transfer learning for plant disease diagnosis, demonstrating how pre-trained models can improve the accuracy and reliability of disease detection in varied environmental conditions, thus enhancing crop management practices (Chen et al., 2020). A fourth study applies transfer learning techniques to detect tomato plant diseases, highlighting the importance of deep learning in early disease detection and control, which supports sustainable agriculture practices (Abbas A. et al., 2021). Finally, the fifth paper introduces a method for classifying pests in crops using deep convolutional neural networks, offering a solution for pest management, which is crucial for protecting crops from harmful pests and ensuring better yields (Thenmozhi and Reddy, 2019). These contributions collectively highlight the pivotal role of deep learning in advancing agricultural technologies, focusing on disease and pest detection, and improving the efficiency and sustainability of crop management (Table 1).

**Table 1:** Bibliometric and content attributes of representative documents.

Title	Year	Authors	Country/regions	Publication/source titles	Cited reference count	Keywords
Identification of rice diseases using deep convolutional neural networks	2017	Lu et al. (2017)	China	NEUROCOMPUTING	485	Identification of rice diseases; Convolutional neural networks; Deep learning; Image recognition
Fruit detection for strawberry harvesting robot in non-structural environment based on Mask-RCNN	2019	Yu et al. (2019)	China	COMPUTERS AND ELECTRONICS IN AGRICULTURE	462	Mask-RCNN; Non-structural environment; Fruit detection; Instance segmentation; Picking point
Using deep transfer learning for image-based plant disease identification	2020	Chen et al. (2020)	China	COMPUTERS AND ELECTRONICS IN AGRICULTURE	446	Plant disease identification; Deep learning; Convolution neural networks; Transfer learning; Image classification
Tomato plant disease detection using transfer learning with C-GAN synthetic images	2021	Abbas A. et al. (2021)	India	COMPUTERS AND ELECTRONICS IN AGRICULTURE	293	Deep learning; Tomato plant disease detection; Conditional Generative Adversarial Network; Data augmentation; Pre-trained DesnseNet121 network; Synthetic Images
Crop pest classification based on deep convolutional neural network and transfer learning	2019	Thenmozhi and Reddy (2019)	India	COMPUTERS AND ELECTRONICS IN AGRICULTURE	276	Insect classification; Field crops; Convolutional neural network; Deep learning; Transfer learning

### 2.2 Analysis method

Bibliometric analysis is a systematic, quantitative approach used to summarize and evaluate research publications. It involves four primary steps: sampling, recording entries, cataloguing, and metrics. The first three steps focus on standardizing data, including titles, authors, keywords, publication dates, methodologies, and citation counts. The final step, metrics, extracts valuable insights to guide future research. This process is greatly facilitated by software tools such as *VOSviewer* (version, 1.6.16) and *CiteSpace* (version, 6.3.R1). *VOSviewer*, developed by the Centre for Science and Technology Studies (CWTS) at Leiden University, specializes in visualizing scientific knowledge and analyzing undirected networks. *CiteSpace*, created by Professor Chen Chaomei at Drexel University, is used for citation analysis and identifying potential knowledge within scientific research. Both tools help uncover trends, research hotspots, and the evolution of scientific fields (Bouguettaya et al., 2022).

The knowledge graph for this study was constructed using *VOSviewer* and *CiteSpace*, two prominent tools in bibliometric analysis. *VOSviewer* was used for visualizing the relationships between key terms, authors, and journals. The tool applied co-occurrence analysis to generate term maps, where terms frequently appearing together in the dataset were grouped into clusters. These clusters represent research topics or themes within the field of AI and agriculture. The visual representation provided by *VOSviewer* highlighted the major research areas in crop monitoring, such as disease detection and pest management, as well as the dominant methodologies like Convolutional Neural Networks (CNNs) and remote sensing integration. *CiteSpace* was employed to conduct citation analysis, identifying influential papers, key authors, and significant trends in the field over time. *CiteSpace*’s citation burst detection feature was particularly useful in identifying rapidly emerging topics, allowing for the identification of key shifts in research focus. The software also provided a visualization of the collaborative networks between authors and institutions, enabling us to examine how knowledge flows and research collaborations have evolved in the AI-driven agricultural field.

By combining these two tools, we were able to not only extract key research themes and identify gaps in the literature but also understand the temporal evolution and collaborative dynamics within the field. The methodology allowed us to construct a comprehensive knowledge graph that reveals how AI and deep learning technologies are shaping agricultural research and practice.

This paper employs two primary research methods: bibliometric analysis and knowledge mapping analysis, to explore the analysis of agricultural information identification and monitoring. Various bibliometric tools, including WOS, Scopus, VOSviewer, and SCImago Graphica, are utilized to process data and analyze key aspects such as publication times, contributions, and highly cited works. VOSviewer is particularly useful for co-occurrence analysis and the visualization of research hotspots, while CiteSpace is applied to examine research cooperation, identify emerging keywords, and predict future trends (Iyortsuun et al., 2023; Rubbens et al., 2023). By combining these software tools, the study maximizes their respective strengths, offering a comprehensive view of the research field’s development and providing valuable insights into the ongoing evolution of scientific knowledge in this area.

## 3 Results

### 3.1 Bibliometric analysis

#### 3.1.1 Statistics of documents publication time

**Figure 2:**
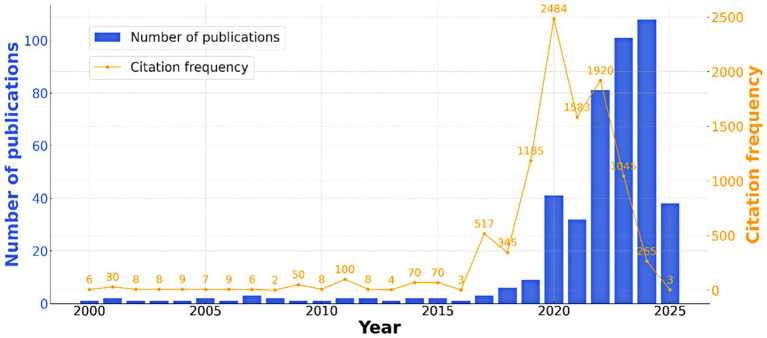
Annual scholarly productivity and citation impact over two decades (2000–2022).

During the analysis of the application of machine learning in intelligent crop recognition, the data sourced from the WOS database was used to track the trends in publication and citation frequency over the years. The chart in Figure 2 illustrates these trends, with the blue bars representing the number of publications in each year and the orange line representing the citation frequency. The data reveals several key insights: In the early years (2000–2010), the number of publications remained low, with fewer than 50 papers published annually, reflecting the initial phase of research in this area. From 2011 to 2017, there was a sharp increase in both publications and citations, peaking in 2012 with over 90 citations. This period marks the rapid development and advancement of machine learning techniques in crop recognition, as researchers placed greater emphasis on enhancing the accuracy and efficiency of algorithms. The period from 2018 to 2022 shows a steady rise in the number of publications, with a particularly significant surge in 2023 and 2024, where publication numbers exceeded 80, reflecting the growing interest and advancements in the field. Citation frequency also peaked during this period, indicating the increasing impact of this research. The overall trend highlights a growing recognition of the potential of machine learning in agriculture, with a clear upward trajectory in both research output and influence. This development marks the shift toward more advanced, AI-driven systems for intelligent crop identification. It can be concluded that the research on the application of machine learning in intelligent crop image recognition has generally progressed through three key research stages over the past two decades, as reflected by the trends in publication numbers and citation frequencies (Figure 2).

#### 3.1.2 Stage 1: exploratory and experimental stage (2000–2010)

The first stage, from 2000 to 2010, represents the initial foray into machine learning techniques applied to crop image recognition, with a focus on early experimentation and the testing of various algorithms for crop disease and pest identification. During this period, researchers primarily relied on traditional image processing methods combined with machine learning algorithms to address the challenges of crop monitoring. Key studies focused on the early development of machine learning systems for the classification of crop diseases based on visible symptoms. Works such as Loss et al. (2009) marked foundational contributions, introducing the basic concepts of image recognition systems for agriculture. Although the technology was still in its infancy, these early efforts led to the development of the first generation of crop disease recognition systems using computer vision techniques, which laid the groundwork for the later integration of more advanced machine learning models. This exploratory stage was characterized by the application of basic classifiers, such as support vector machines (SVMs) and decision trees, for crop image analysis (Loss et al., 2009). The research in this phase was limited by the computational resources available at the time and the relatively small and simple datasets used for training models. However, these early studies played a crucial role in demonstrating the potential of machine learning in agriculture and established a solid foundation for the future development of more complex deep learning models. Despite the relatively low number of publications and limited citation counts in this stage, the research set the stage for the transition to more sophisticated machine learning techniques in subsequent years.

#### 3.1.3 Stage 2: technology maturation stage (2011–2017)

Between 2011 and 2017, the field of crop image recognition through machine learning experienced significant technological maturation. During this period, there was a marked shift from traditional machine learning methods to more advanced deep learning techniques, particularly convolutional neural networks (CNNs), which became central to crop disease and pest detection tasks. The use of CNNs allowed researchers to automate and refine the process of feature extraction from crop images, leading to substantial improvements in classification accuracy. This stage also witnessed the increasing use of large-scale image datasets, including those captured by UAVs and satellites, which provided a more comprehensive and detailed view of crop conditions across various environments. Studies like Lu et al. (2017), Rakun et al. (2011), and Lu et al. (2015) exemplify the growing sophistication of the models used in crop image recognition. Researchers began to explore more complex applications, such as the detection of specific crop diseases and the classification of pest infestations, using deep learning models trained on a diverse set of crop images. This period also saw significant research into the integration of multi-source data, such as the combination of satellite imagery with field-collected images to improve the robustness and reliability of crop disease detection systems (Lu et al., 2015). The introduction of transfer learning techniques enabled researchers to leverage pre-trained models on large image datasets, significantly improving the efficiency and effectiveness of the models used in agricultural applications. The number of publications increased substantially during this period, with research expanding to cover a wider array of crop species and environmental conditions. Moreover, the citation frequency for these studies grew rapidly, reflecting the increasing importance of machine learning in the field of precision agriculture. By the end of this stage, machine learning models had reached a level of maturity that allowed for real-world applications in agriculture, paving the way for the next stage of development in crop image recognition.

#### 3.1.4 Stage 3: artificial intelligence phase (2018–2022)

The period between 2018 and 2022 represents the rise of artificial intelligence (AI), with deep learning and machine learning models emerging as the leading technologies for intelligent crop image recognition. During this phase, AI-driven systems for crop monitoring and disease detection gained widespread adoption, leading to a significant surge in both research output and citation rates in the field. The application of machine learning in agriculture shifted towards automated, real-time crop monitoring systems that were capable of handling large volumes of data and providing more accurate predictions. AI-driven models, particularly convolutional neural networks (CNNs) and advanced neural networks, became central to crop disease detection, pest management, and phenological stage monitoring. Studies such as Saranya et al. (2021), Sharma et al. (2022), and Li et al. (2022) highlight the increasing reliance on AI for real-time monitoring of crops. The integration of UAV-based imagery and machine learning models allowed for the collection of high-resolution data on crop health, enabling more precise detection of diseases, nutrient deficiencies, and pests. This period also saw the development of more sophisticated AI systems that combined instance and semantic segmentation with deep learning for improved crop disease and pest detection, as demonstrated in studies like Tassis et al. (2021) and d’Andrimont et al. (2022). Moreover, the application of AI extended to various aspects of precision agriculture, such as the classification of nutrient deficiencies in apple trees (Kumar et al., 2022) and the development of automatic systems for pest detection and crop yield prediction. The growing use of AI in agriculture has led to the emergence of cloud-based platforms that allow for the rapid processing of crop images and the integration of these platforms with existing agricultural management systems. The rapid advancements in this phase have made it clear that AI will play a central role in the future of agriculture, with AI-driven systems providing real-time, high-precision solutions for crop monitoring and management (De Angelis et al., 2023; Loeffler et al., 2024; Liu S. et al., 2025). As research continues to evolve, the combination of AI with other emerging technologies, such as robotics and the Internet of Things (IoT), is set to further transform the agricultural industry.

### 3.2 Contribution analysis

In the realm of machine learning applications for crop image recognition, several authors have made pivotal contributions, shaping the field with their innovative approaches. Zhang (Y) has significantly advanced the automation of crop ripeness detection, particularly in jujube fruits, by developing AGHRNet: An attention ghost-HRNet for confirmation of jujube fruit ripening stage. This attention-based deep learning model, with its exceptional performance, has greatly enhanced precision in crop management systems, contributing extensively to the recognition of fruit ripeness and disease detection (Zheng et al., 2023). Following closely, Chen (C) has made important strides in improving crop disease recognition by addressing the challenges posed by occluded images. His work on HOB-CNN: Hallucination of occluded branches with convolutional neural networks introduced methods to handle imperfect data, making crop disease detection more reliable even in the presence of visual obstructions (Chen et al., 2023). Meanwhile, Saraswat (D) has focused on optimizing deep learning models for large-scale agricultural settings, particularly in crop disease detection. His contributions, including Performance of deep learning models for classification and detection of crop diseases, have greatly improved detection accuracy, making real-time agricultural monitoring more efficient and actionable (Ahmad et al., 2021). He (Y) has significantly advanced crop image recognition through his work on Object detection with attribute tagging task: A novel approach for crop image recognition. His research integrates object detection with attribute tagging, improving crop classification accuracy and enabling systems to capture additional contextual information, such as growth stages. This approach enhances real-time crop monitoring, contributing to more efficient and scalable agricultural management systems (Liu D. et al., 2025). Lastly, Fu (LS) has contributed to the field by introducing new segmentation techniques for crop disease detection. In Deep learning based segmentation for automated crop disease detection, Fu’s innovative methods have improved the precision of disease detection, particularly in tree fruits and vegetables, pushing the boundaries of crop image recognition (Majeed et al., 2020). Together, these authors have significantly refined machine learning models for crop recognition and disease management, with each making unique contributions that continue to propel the field forward, offering solutions for more accurate, real-time, and scalable crop monitoring in agriculture. The work of these five authors shares a common focus on advancing machine learning models for crop image recognition, particularly in disease detection and classification, yet each brings a unique perspective, with some emphasizing deep learning segmentation techniques, others integrating multi-source data, and a few focusing on the automation and real-time applications of crop monitoring systems. The top 5 authors and their representative contributions are listed in Table 2.

**Table 2:** Leading contributors by publication output (Top 5 authors).

Authors	Post volume	Masterpiece	Representative contribution	Index
Zheng et al. (2023)	9	AGHRNet: An attention ghost-HRNet for confirmation of catch-and-shake locations in jujube fruits vibration harvesting	The development of an intelligent jujube fruit harvesting device is a critical step in achieving the whole mechanization process. Catch-and-shake harvesting, as an efficient and stable vibration harvesting method, has widely been used to save labor and improve harvesting efficiency in large-scale jujube orchards. However, existing catch-and-shake harvesters still rely heavily on the operator’s naked eyes to determine the shaking position, which is subjective, highly inefficient and highly labor.	546
Chen et al. (2023)	7	HOB-CNN: Hallucination of occluded branches with a convolutional neural network for 2D fruit trees	Orchard automation has attracted the attention of researchers recently due to the shortage of global labor force. To automate tasks in orchards such as pruning, thinning, and harvesting, a detailed understanding of the tree structure is required. However, occlusions from foliage and fruits can make it challenging to predict the position of occluded trunks and branches. This work proposes a regression-based deep learning model, Hallucination of Occluded Branch Convolutional Neural Network (HOB-CN).	328
Ahmad et al. (2021)	6	Performance of deep learning models for classifying and detecting common weeds in corn and soybean production systems	Knowing precise location and having accurate information about weed species is a prerequisite for developing an effective site-specific weed management (SSWM) system. Due to the effectiveness of deep learning techniques for vision-based tasks such as image classification and object detection, its use for discriminating between weeds and crops is gaining acceptance among the agricultural research community. However, limited studies have used deep learning for identifying multiple weeds in a singl.	183
Liu D. et al. (2025)	6	Object detection with attribute tagging task: Model design and evaluation on agricultural datasets	Object attributes provide valuable information for numerous practical applications and hold potential to augment the generalization capabilities of object detection model. However, research into instance-level attribute recognition has been relatively scant, particularly concerning task optimization and its integration for comprehensive model performance enhancement. This paper introduces the Object Detection with Attribute Tagging (OD-AT) task, designed to detect objects while simultaneously id.	160
Majeed et al. (2020)	5	Deep learning based segmentation for automated training of apple trees on trellis wires	Trellised fruiting-wall training systems are becoming standard for modern apple orchards due to their high fruit yield and quality, and their suitability to robotic operations in pruning and harvesting. In a common practice of training young apple trees to a trellis-trained canopy system in PNW region of US, trees branches are manually selected and then tied to horizontal trellis wires in 6 or 7 tiers. As manual training of apple trees to these modern orchard architectures is becoming challenging.	189

**Figure 3:**
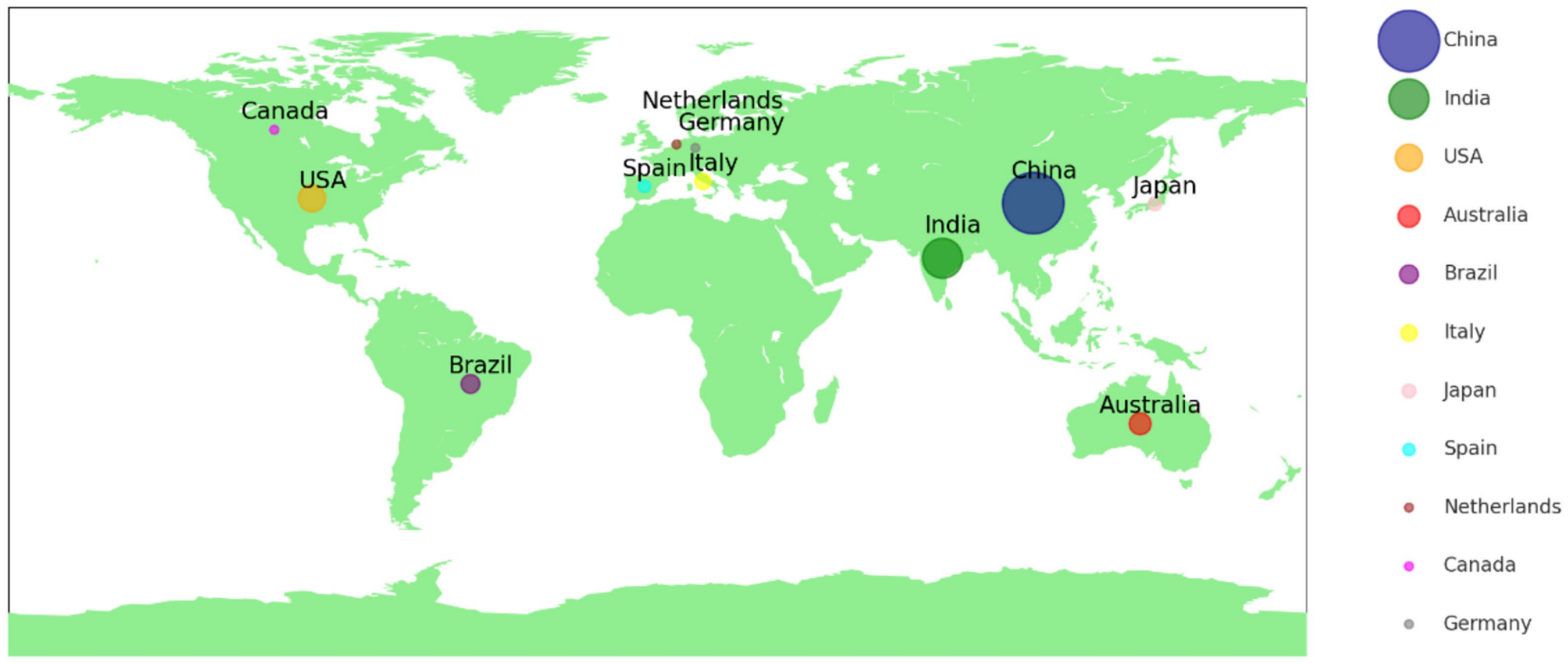
Heatmap of document affiliations by geographic region (Top 11).

To enhance the visual representation of the geographic distribution of publications by country or region, this study first saves the merged and de-duplicated data from the WOS database in a standardized *.txt format. The word frequency threshold is then set to 10 in VOSviewer, allowing for the identification of countries or regions with significant contributions to the field. After processing, the data is saved in *.gml format and imported into Scimago to create a geographical visualization of document distribution, with appropriate map decorations. Figure 3 illustrates the distribution of countries leading globally in crop image recognition research. A total of 11 countries have published more than 10 documents. An analysis of the top five contributing countries reveals notable patterns in the global distribution of crop image recognition research. China leads the field with 208 publications, reflecting its dominant role and the active, extensive research community engaged in this area. India follows with 88 publications, marking its significant contribution to agricultural research, particularly in precision farming and remote sensing. The USA ranks third with 40 publications, demonstrating substantial involvement in crop image recognition, though at a smaller scale compared to China and India. Australia (26 publications) and Brazil (19 publications) round out the top five, indicating growing research efforts, especially in precision agriculture and related technologies. Overall, the top five countries emphasize the prominent positions of China and India in advancing crop image recognition research, with the USA and other nations contributing significantly, though on a relatively smaller scale.

**Figure 4:**
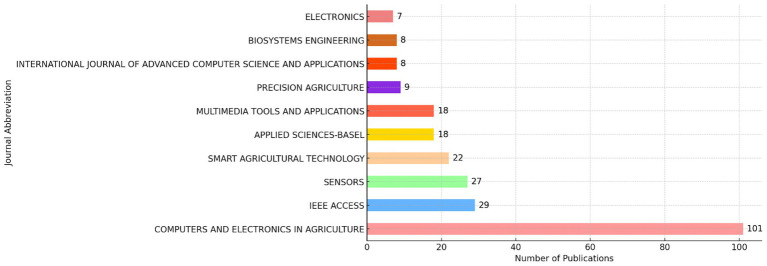
Publication volume across leading journals (Top 10).

After synthesizing the data obtained from WOS, a total of 35 journals were identified, with 12 publishing more than three documents. This suggests significant interest in the research area of crop image recognition. Figure 4 displays the top ten journals. The top three journals in terms of publication count are COMPUTERS AND ELECTRONICS IN AGRICULTURE (109 publications), IEEE ACCESS (29 publications), and SENSORS (27 publications), with corresponding h-index values of 12, 9, and 8, respectively. The top three journals in crop image recognition research, COMPUTERS AND ELECTRONICS IN AGRICULTURE (109 publications), IEEE ACCESS (29 publications), and SENSORS (27 publications), focus on different aspects of the field. COMPUTERS AND ELECTRONICS IN AGRICULTURE primarily publishes papers on computational methods and electronics in agriculture, with a strong emphasis on precision farming, remote sensing, and automation systems for crop monitoring. This journal has seen a consistent increase in publications, particularly from 2010 onwards, as studies combining machine learning and image processing in agricultural applications have grown. In contrast, IEEE ACCESS accepts a broader range of interdisciplinary papers, focusing on the integration of computer science, electronics, and agricultural engineering, especially in developing advanced algorithms for crop image analysis. This journal has experienced a notable rise in publications over the last 5 years, reflecting the growing influence of AI and big data in crop image recognition. Lastly, SENSORS publishes research centered around sensor technologies, particularly in remote sensing and the use of hyperspectral and multispectral imaging for crop health monitoring. Publications in this journal have gradually increased since 2015, as sensor technologies have advanced, enabling more widespread application in agricultural monitoring. Together, these journals reflect a strong shift toward AI, machine learning, and advanced sensor technologies in crop image recognition, with an upward trend in these areas in recent years.

### 3.3 Analysis of highly cited documents

These five papers have attracted significant attention in the field of crop image recognition due to their application of advanced machine learning techniques, particularly deep learning, which have significantly enhanced the accuracy and efficiency of agricultural monitoring systems. A common theme across these papers is the integration of cutting-edge neural network models, such as deep convolutional networks (CNNs) and improved versions of YOLO, to address the challenges of crop detection, disease identification, and fruit harvesting automation. The adoption of techniques like transfer learning has further contributed to the high impact of these papers, as it enables models to generalize well across different crops and environments, even with limited data. The focus on real-time applications, such as automated fruit harvesting and disease detection, resonates with the growing demand for precision agriculture, where efficient, cost-effective, and scalable solutions are paramount. Moreover, the timing of these papers aligns with advancements in sensor technologies, making them even more relevant as the agricultural industry increasingly turns to remote sensing and AI-based tools for monitoring crop health. This convergence of deep learning, data-driven solutions, and agricultural needs has made these papers essential references in the field, driving innovation and inspiring further research in crop image recognition and automation (Table 3). In summary, these papers are highly cited for addressing critical issues in modern agriculture—crop detection, disease identification, and automation—while utilizing advanced machine learning techniques such as deep learning and transfer learning, which enhance their relevance in the field of crop image recognition and precision agriculture.

**Table 3:** Citation analysis of highly cited literature.

References	Cite frequency	Year	Title	Publication type	Research contents
Qi et al. (2022)	916	2022	An improved YOLOv5 model based on visual attention mechanism: Application to recognition of tomato virus disease	Article	Traditional target detection methods cannot effectively screen key features, which leads to overfitting and produces a model with a weak generalization ability. In this paper, an improved SE-YOLOv5 network model is proposed for the recognition of tomato virus diseases. Images.
Yu et al. (2019)	750	2019	Fruit detection for strawberry harvesting robot in non-structural environment based on Mask-RCNN	Article	Deep learning has demonstrated excellent capabilities for learning image features and is widely used in image object detection. In order to improve the performance of machine vision in fruit detection for a strawberry harvesting robot, Mask Region Convolutional Neural Network.
Wu et al. (2022)	635	2022	Detection of Camellia oleifera Fruit in Complex Scenes by Using YOLOv7 and Data Augmentation	Article	Rapid and accurate detection of Camellia oleifera fruit is beneficial to improve the picking efficiency. However, detection faces new challenges because of the complex field environment. A Camellia oleifera fruit detection method based on YOLOv7 network and multiple data augmentation.
Lu et al. (2017)	444	2017	Identification of rice diseases using deep convolutional neural networks	Article	The automatic identification and diagnosis of rice diseases are highly desired in the field of agricultural information. Deep learning is a hot research topic in pattern recognition and machine learning at present, it can effectively solve these problems in vegetable.
Chen et al. (2020)	347	2020	Using deep transfer learning for image-based plant disease identification	Article	Plant diseases have a disastrous impact on the safety of food production, and they can cause a significant reduction in both the quality and quantity of agricultural products. In severe cases, plant diseases may even cause no grain harvest entirely.

### 3.4 Knowledge mapping analysis

#### 3.4.1 Analysis of scientific research cooperation

**Figure 5:**
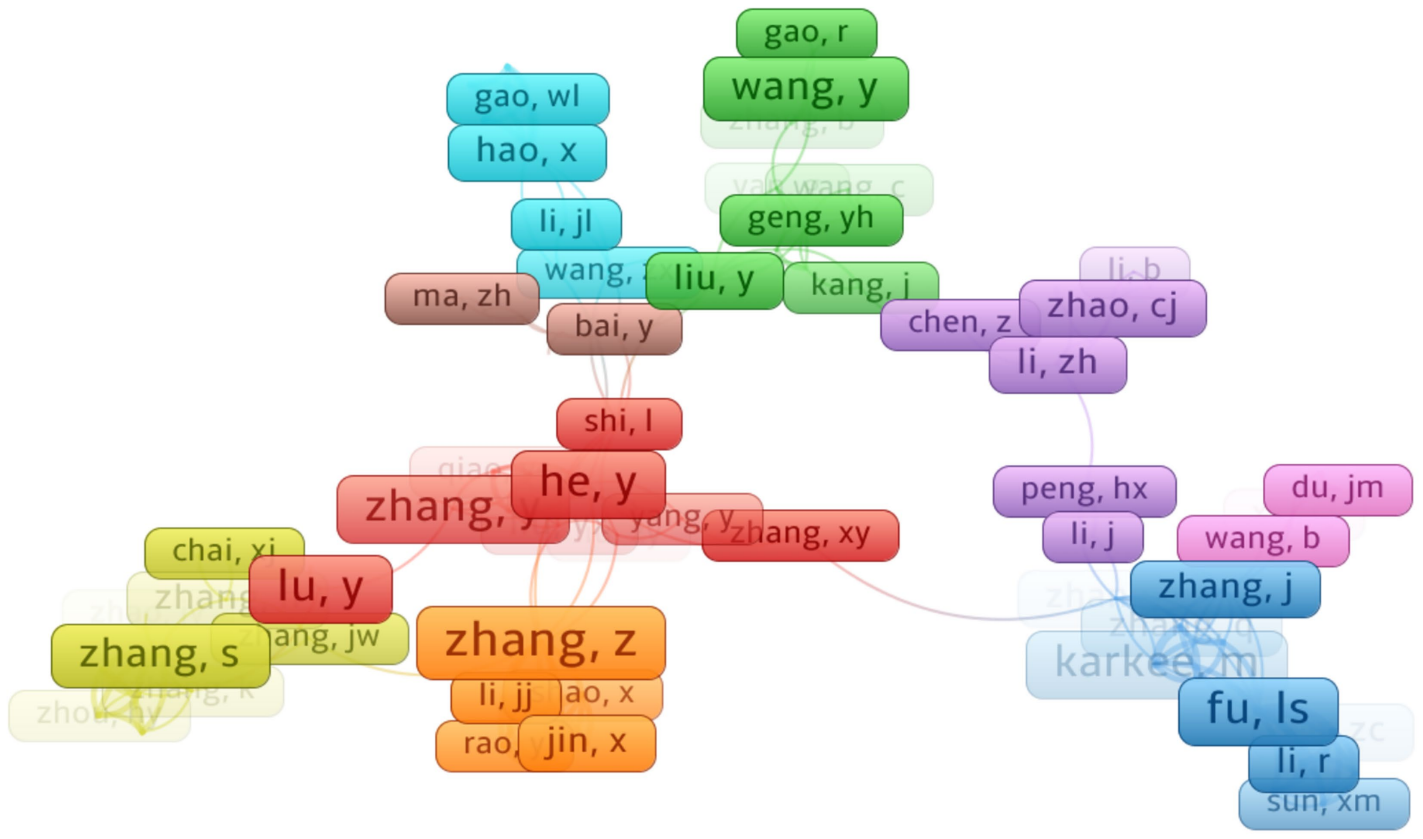
Knowledge graph of academic collaborations.

The author collaboration network was constructed using VOSviewer (version 1.6.16), as illustrated in Figure 5. To enhance clarity, only authors with more than three publications were included in the visualization. The network exhibits a ‘widely distributed but locally concentrated’ pattern, indicating that while research collaborations are widespread, concentration tends to occur around specific core research groups. Among the most influential authors in the field of crop image recognition using machine learning, five key researchers emerge as central figures: He et al. (2024), Wang et al. (2025), Kamei (2023), Sarah et al. (2024), and Zhang Z. et al. (2023). These scholars have made substantial contributions through their extensive collaborative efforts, with He Y. playing a particularly pivotal role in multiple high-impact studies. Wang Y., Karkee. M., Saraswat, and Zhang Z. have also demonstrated strong research activity, further consolidating their influence in this domain. Their collective work has made significant contributions to the advancement of machine learning applications in agricultural image analysis, fostering innovations in crop monitoring, disease detection, and yield prediction. The network visualization highlights a highly interconnected structure among these authors, underscoring their central role in driving the research landscape forward. This dense collaboration pattern emphasizes the critical role of interdisciplinary teamwork in accelerating progress within this rapidly evolving field. Additionally, the visualization reveals evidence of South–South collaboration, further illustrating the global nature of research in this domain and the increasing contributions from developing regions.

**Figure 6:**
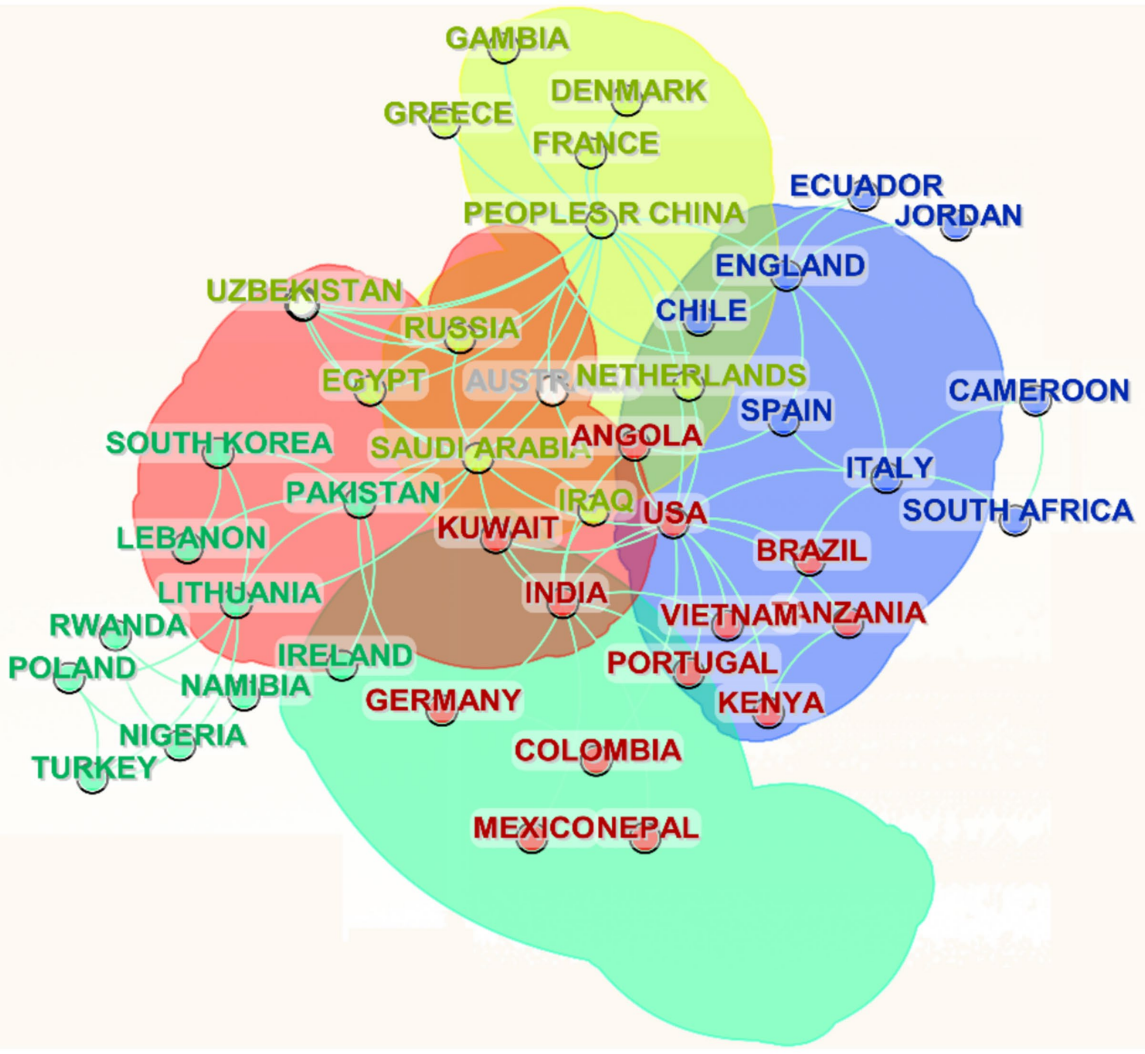
International research collaboration network.

Using CiteSpace, a visualization of international academic collaborations are generated based on the extracted country data (Figure 6). The analysis reveals a robust network of scientific cooperation, with China, India, and the USA emerging as the most frequent collaborators. China, in particular, stands out as the most active participant, engaging in 172 collaborative instances, underscoring its central role in the global research landscape. Other notable contributors include Australia (22 collaborations), Japan (18), and Saudi Arabia (16), reflecting their substantial involvement in academic exchange. Additionally, countries such as Brazil, South Korea, and Italy, though less frequent collaborators, still play meaningful roles in the network. Smaller nations such as Pakistan, Spain, Greece, and Egypt also appear in the dataset, reflecting their involvement in international research efforts despite having lower collaboration counts. The network structure reveals a clear hierarchical pattern, with the USA, China, and India positioned as central hubs, linked to numerous other countries across diverse regions. This pattern highlights the increasingly globalized nature of scientific research, with nations at different stages of economic and scientific development actively participating in cross-border partnerships. Additionally, the data suggests a correlation between a country’s economic development and its research collaboration output, with more economically advanced nations typically taking the lead in global academic cooperation. These observations align with broader trends in the internationalization of science, emphasizing the critical role of collaborative networks in advancing research across disciplines.

**Figure 7:**
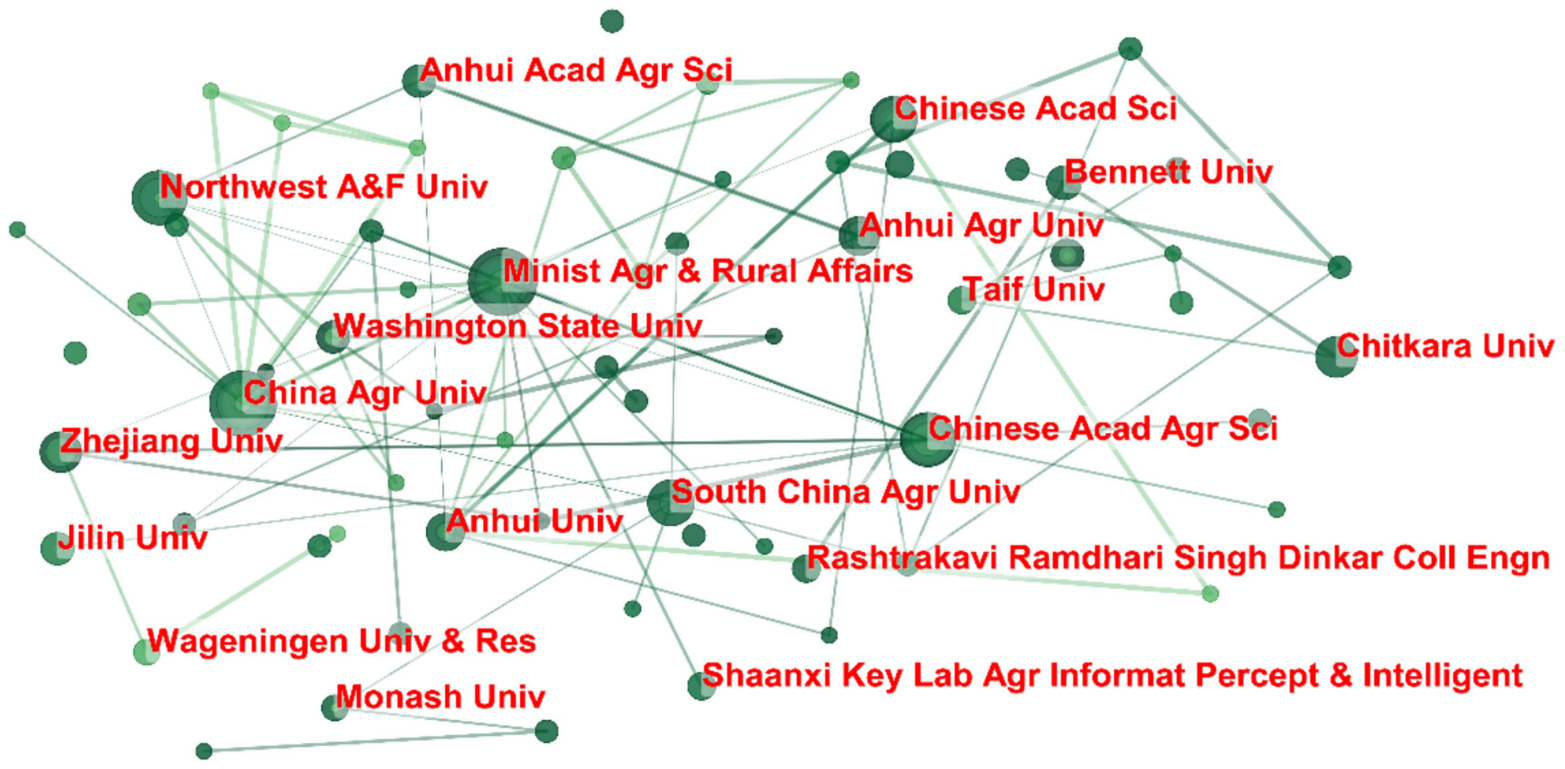
Institutional collaboration network.

The network visualization generated using CiteSpace illustrates the collaborative relationships among academic institutions based on co-authorship or institutional affiliations in the dataset (Figure 7). The graph reveals meaningful patterns of cooperation, with nodes representing institutions and edges indicating collaborative interactions. Key institutions within the network include Anhui Agricultural University, Washington State University, Monash University, Chinese Academy of Agricultural Sciences, and Shaanxi Key Laboratory of Agricultural Information Perception and Intelligent Servicing, which serve as central hubs with multiple connections. These institutions demonstrate a high degree of interconnectivity, suggesting active collaboration in research activities. Meanwhile, other institutions appear more peripherally, reflecting fewer but still significant collaborative ties. The presence of well-connected hubs indicates that institutional cooperation follows a scale-free network structure, where a few highly active institutions dominate the collaboration landscape, while others contribute to a broader but less dense network. This pattern aligns with global trends in scientific collaboration, where leading research institutions often function as central nodes in knowledge exchange and co-authored publications. Further analysis could explore the thematic focus of these collaborations and their impact on research output.

#### 3.4.2 Analysis of research hotspots

**Figure 8:**
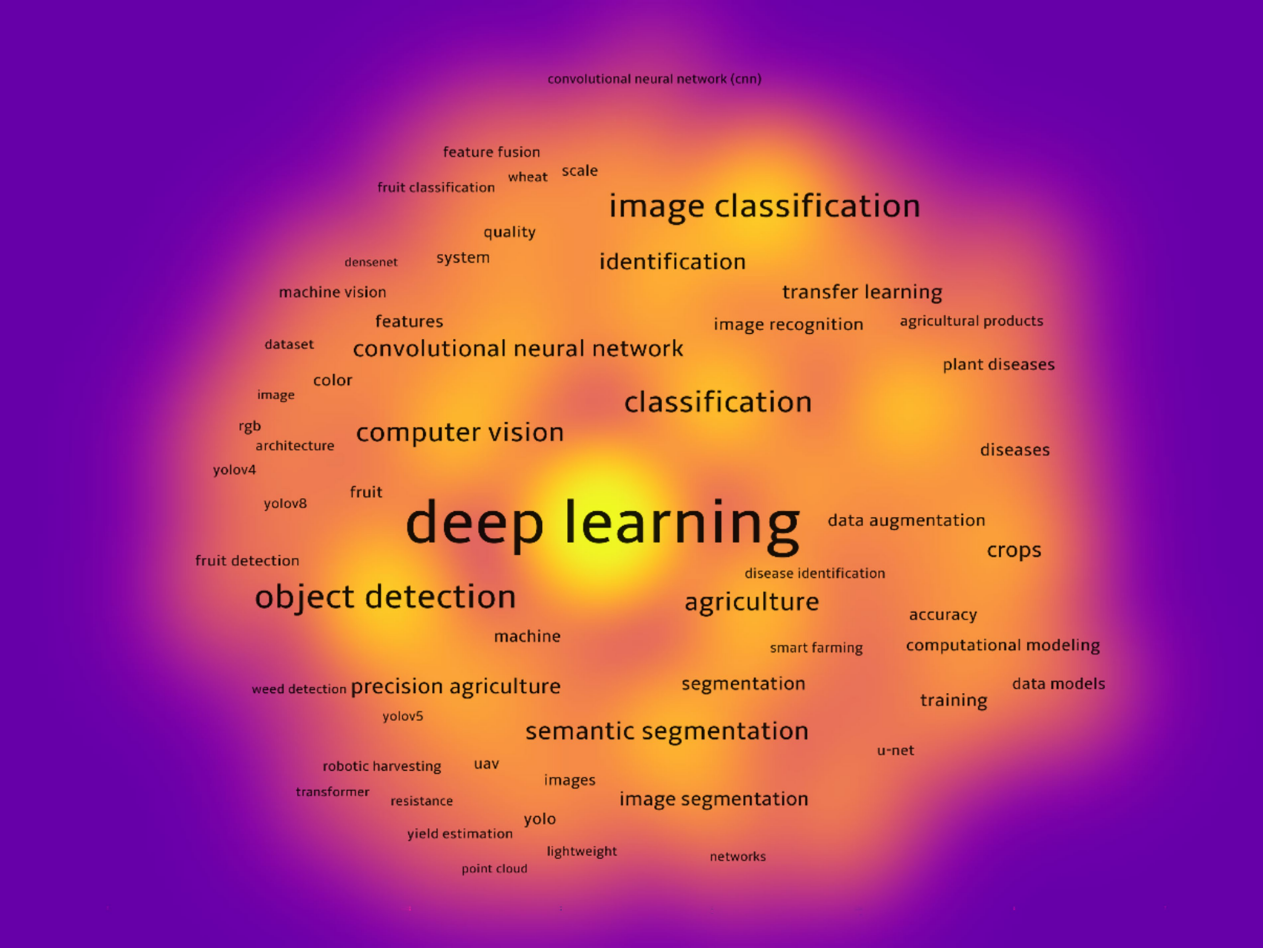
Co-occurrence network of high-frequency keywords.

Based on the provided image, the research hotspots in the field of deep learning applied to agriculture can be summarized as follows (Figure 8). The central theme in the image is “deep learning, “which is the dominant research focus, illustrated by its large node size and central position. Surrounding it are various subtopics, with prominent terms such as “image classification, ““object detection, “and “semantic segmentation, “which emphasize key areas of deep learning applications in agricultural image analysis. The green keyword cluster focuses on “agriculture, “indicating the integration of deep learning with agricultural applications such as crop disease identification, precision agriculture, and agricultural product recognition. The red cluster highlights “object detection” and “fruit detection, “which are essential techniques for recognizing and classifying objects within agricultural imagery. Keywords like “yolo, ““point cloud, “and “rgb architecture” show how deep learning is being used in detecting crops and agricultural products, along with their respective quality assessment. The yellow keyword group, associated with “machine vision, “suggests the importance of visual systems for image recognition and classification in agricultural settings. Other relevant keywords, such as “transfer learning” and “data augmentation, “indicate the use of advanced techniques to enhance model accuracy and adaptability in agricultural applications. In summary, the hot research directions in this field are centered around the application of deep learning to enhance agricultural practices, with a particular focus on image-based classification, object detection, and advanced segmentation methods.

**Figure 9:**
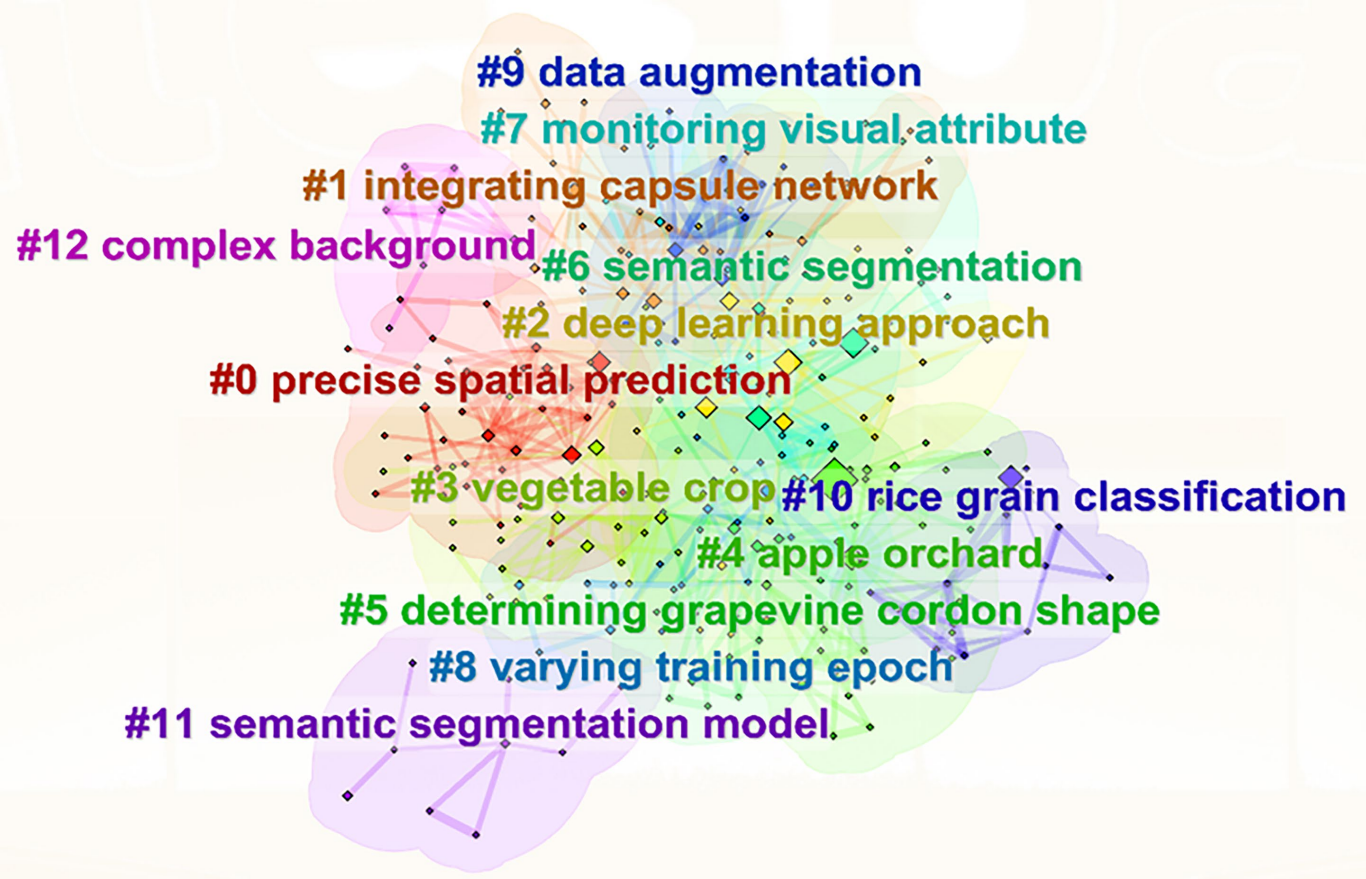
Research clusters identified by CiteSpace.

To further clarify the internal connections among research hotspots and explore their respective research focuses and development directions, CiteSpace (version 6.3.R1) was employed to generate a keyword cluster map based on VOSviewer keyword co-occurrence analysis, as shown in Figure 9. The research in the domain of intelligent image recognition for crops using machine learning can be categorized into 13 distinct clusters, namely: #0 precise spatial prediction, #1 integrating capsule network, #2 deep learning approach, #3 vegetable crop, #4 apple orchard, #5 determining grapevine cordon shape, #6 semantic segmentation, #7 monitoring visual attribute, #8 varying training epoch, #9 data augmentation, #10 rice grain classification, #11 semantic segmentation model, and #12 complex background. The clusters are color-coded and numerically sorted based on their prominence and frequency in the dataset. A larger cluster number indicates a relatively higher publication volume and greater impact in the field. According to the clustering results, these topics can be summarized into four main research directions:

Research on segmentation models and prediction algorithms is represented by clusters such as #0 precise spatial prediction, #1 integrating capsule network, #2 deep learning approach, #6 semantic segmentation, and #11 semantic segmentation model, which collectively form the technical foundation of intelligent image recognition. These clusters emphasize model optimization, the integration of deep learning frameworks (e.g., CNNs and capsule networks), and the importance of achieving pixel-level accuracy in spatial predictions. Clusters #3 vegetable crop, #4 apple orchard, #5 determining grapevine cordon shape, and #10 rice grain classification highlight the application of machine learning and computer vision techniques to different crop species, reflecting a growing interest in developing targeted solutions for specific crop characteristics, growth monitoring, and yield estimation. Clusters #7 monitoring visual attribute, #8 varying training epoch, and #9 data augmentation underscore efforts toward improving training optimization, model generalization, and visual attribute analysis. These clusters stress the importance of robust data preparation and adaptive learning strategies in agricultural environments. Cluster #12 complex background identifies the challenges posed by real-world agricultural conditions, where environmental variables and image noise often affect recognition accuracy, calling for solutions to enhance model resilience in non-ideal or cluttered field conditions.

#### 3.4.3 Analysis of frontier trend

**Figure 10:**
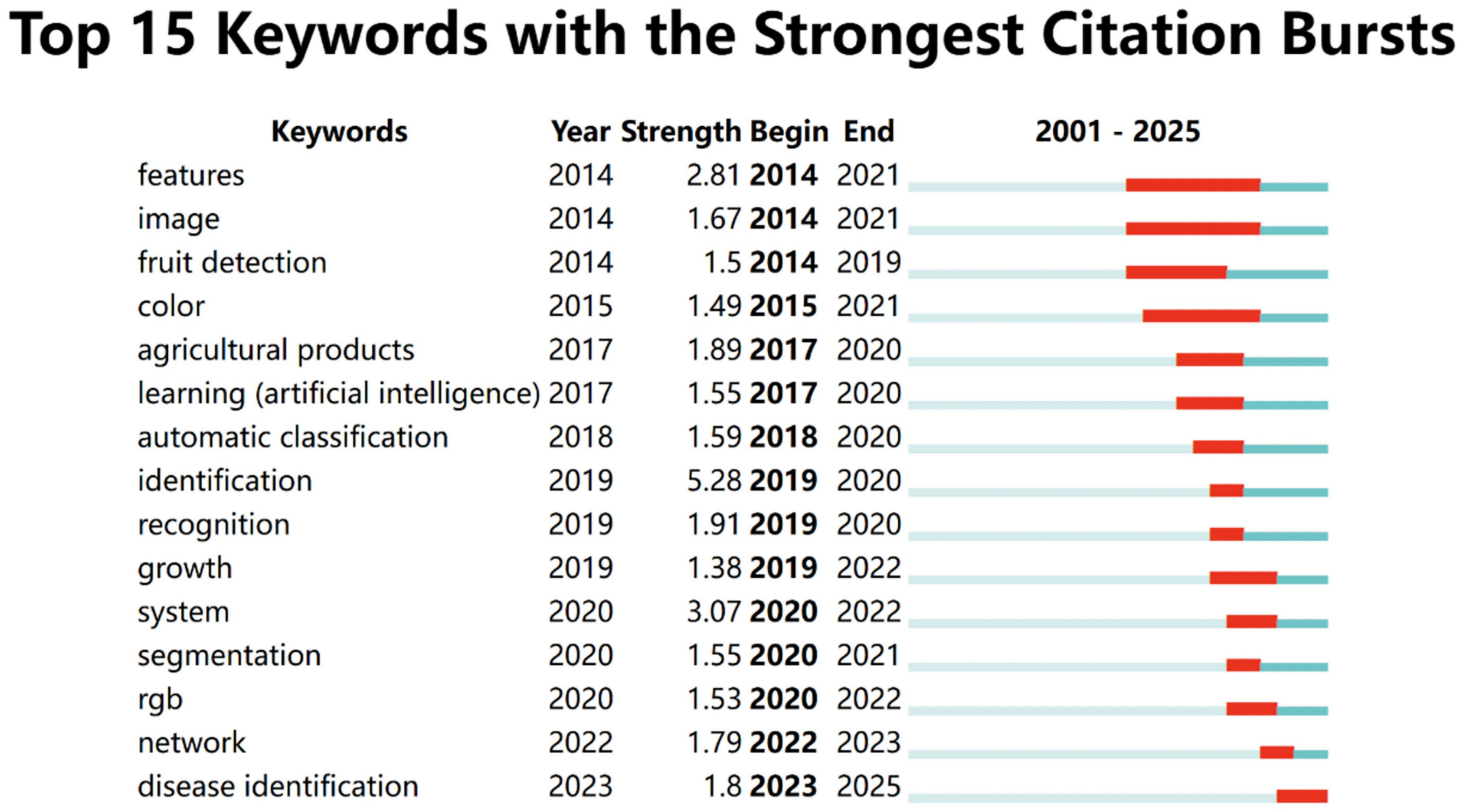
Keyword mutation (burst detection) analysis.

To further investigate the temporal dynamics of research trends in the field of machine learning-based crop image recognition, a burst detection analysis was conducted using CiteSpace. As shown in Figure 10, the top 15 keywords with the strongest citation bursts between 2001 and 2025 reveal significant shifts in scholarly focus over time. The keyword “system” demonstrates the highest burst strength (3.07), primarily occurring between 2020 and 2022, indicating a strong recent interest in integrated image recognition systems in agriculture.

Early research emphasis, as indicated by keywords such as “features,” “image,” “fruit detection,” and “color,” focused on fundamental image processing techniques and object-level identification, with bursts starting as early as 2014 and ending around 2021. These foundational studies laid the groundwork for subsequent advances in deep learning and classification. From 2017 onwards, attention shifted toward “agricultural products,” “learning (artificial intelligence),” and “automatic classification,” reflecting the rapid integration of AI and machine learning methods in agricultural contexts. Notably, “identification” recorded an exceptionally high burst strength (5.28) in 2019, although it was short-lived, indicating a temporary but intense focus on classification accuracy and methodology. Recent years (2020–2025) highlight the rise of more complex themes such as “segmentation,” “network,” and “disease identification,” signifying the progression from basic detection tasks to more sophisticated applications like semantic segmentation and crop disease diagnosis using convolutional neural networks and deep feature extraction. The emergence of “disease identification” in 2023, which remains ongoing, suggests a current and rapidly evolving research frontier aimed at practical, disease-specific applications of intelligent image recognition systems. These burst trends not only reveal the historical development of the field but also emphasize emerging research priorities that align with real-world agricultural needs and technological capabilities.

**Figure 11:**
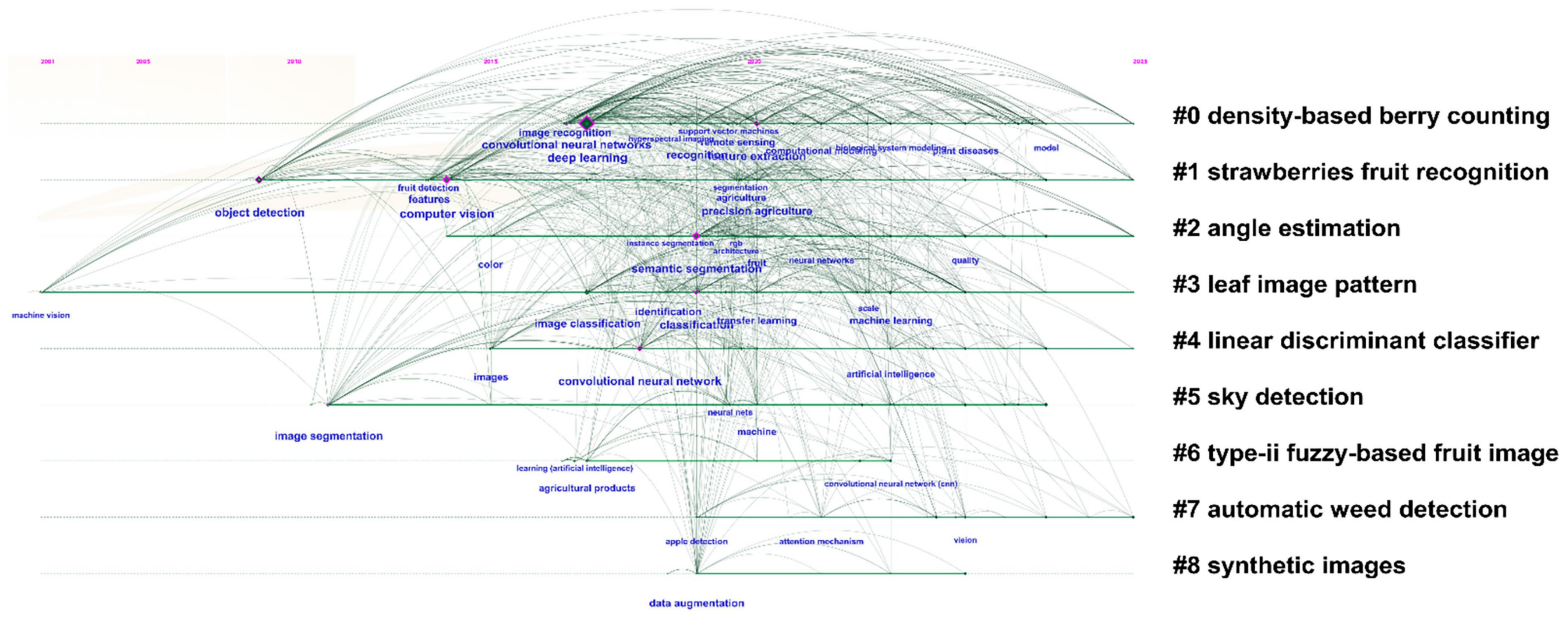
Topic evolution timeline generated by LSR (Logarithmic Successive Ratio) algorithm.

The timeline chart, generated using *CiteSpace* (version 6.3. R1), presents a comprehensive visualization of the evolution of research in the domain of machine learning-driven intelligent recognition for agricultural crop analysis. Figure 11 maps the development trajectory of key research topics over time, based on clusters of keywords derived from a broad selection of scholarly articles. These keywords are arranged along a timeline to capture their emergence, co-occurrence, and frequency over various research phases. In particular, the chart highlights the central role of deep learning and computer vision techniques, with a marked emphasis on areas such as “density-based berry counting, “strawberries fruit recognition,” and “leaf image pattern analysis,” which appear as early and significant focal points. Early studies predominantly revolved around the identification and classification of individual crop types, employing simpler recognition algorithms and methodologies. However, as research has progressed, these themes have evolved to encompass more advanced areas such as “automatic weed detection,” “sky detection,” and “synthetic image generation,” reflecting a shift toward more complex and multifaceted applications in agricultural analysis.

The increasing complexity of these research topics is also evidenced by the expansion of the timeline, with certain clusters such as “convolutional neural networks” and “image segmentation” enduring for extended periods, signaling a growing research consensus and the emergence of increasingly sophisticated computational models. These advancements not only signify technical growth but also illustrate a broader application of machine learning techniques across various sub-domains, ranging from crop identification to environmental factors and agricultural productivity management. Moreover, the chart also offers insights into how the intersection of various disciplines, such as computer vision, data segmentation, and environmental monitoring, has been instrumental in shaping the future of agricultural research. As machine learning models continue to be refined, their integration with agricultural practices is expected to enhance crop management, yield prediction, and pest detection capabilities, thereby contributing to more sustainable farming practices globally.

### 3.5 Main research subfields

**Figure 12:**
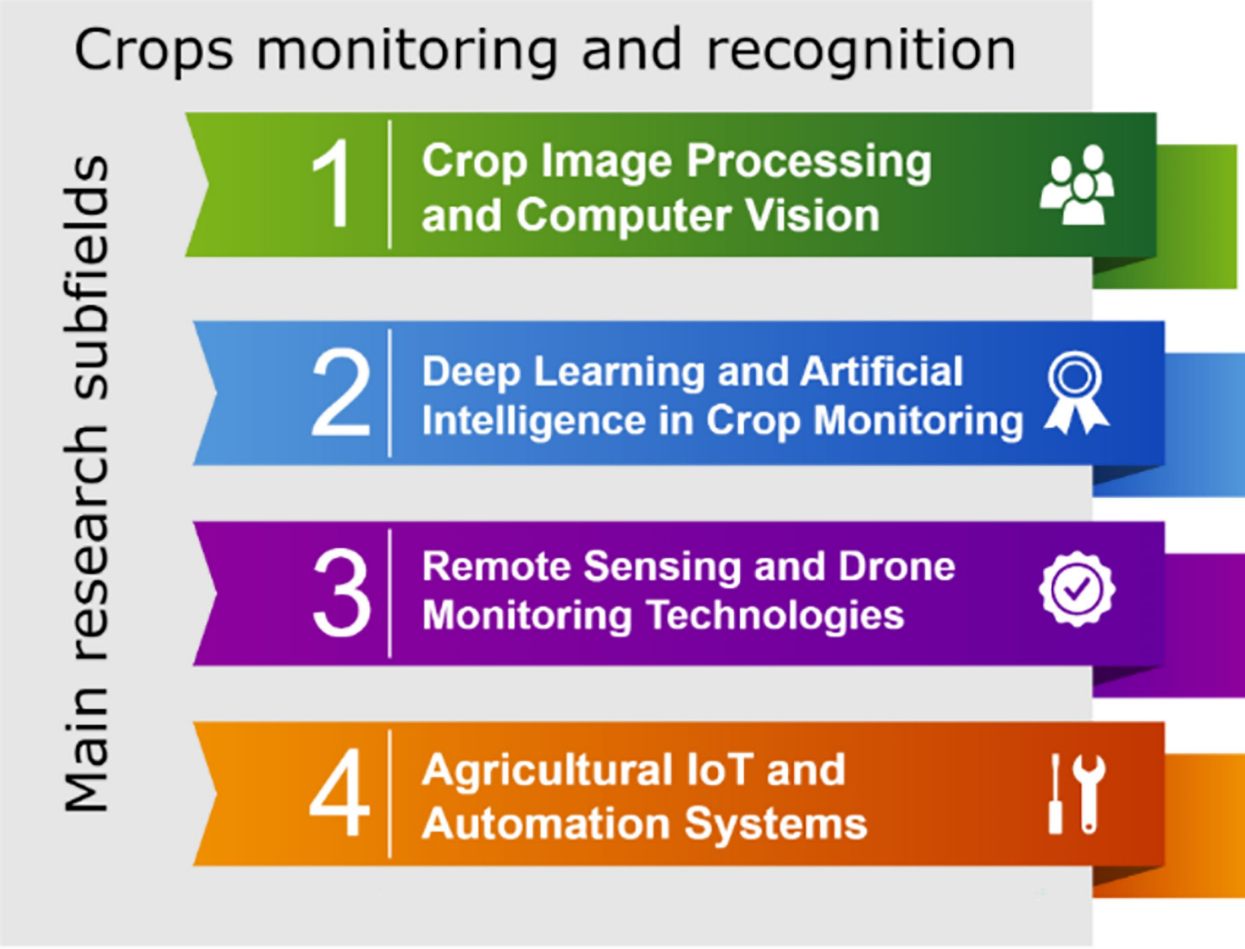
Hotspot detection: fast-growing vs. saturated research areas.

With the advancement of agricultural technologies, crop recognition and monitoring have become essential areas of study in modern agricultural management. The following four subfields have seen widespread application in crop monitoring and recognition (Figure 12).

#### 3.5.1 Crop image processing and computer vision

Crop image processing and computer vision have become foundational tools in the automated monitoring of crops, particularly for disease detection, growth stage classification, and environmental condition assessment. Computer vision systems have been applied to evaluate corn kernel damage, providing crucial insights into yield prediction. Texture analysis methods have been employed to detect fruits, facilitating the automation of fruit harvesting (Chaivivatrakul and Dailey, 2014). Multi-view imaging techniques have also been utilized to characterize maize tassel traits, offering essential data for crop breeding and growth monitoring (Lu et al., 2015). A deep learning-based convolutional neural network (CNN) system for apple segmentation achieved high-precision detection (87.3%) with fast processing (55 ms) in complex environments (Kong et al., 2024). A model using Spatial Pyramid Matching (SPM) combined with Support Vector Machine (SVM) reached an accuracy of 98.15% for apple classification (Ismail et al., 2018). Multi-class fruit detection using region-based CNNs achieved a miss rate as low as 3.77%, outperforming traditional sliding window methods (Tang et al., 2023). Moreover, image classification techniques have been used to detect specific plant parts, such as grapevine buds, aiding in the management of crop health and growth stages (Pérez et al., 2017).

#### 3.5.2 Deep learning and artificial intelligence in crop monitoring

In the field of deep learning and artificial intelligence, these technologies have significantly transformed crop monitoring, especially in the areas of disease identification, pest control, and crop classification. Deep convolutional neural networks (CNNs) have been applied to detect and classify diseases in rice crops, providing farmers with real-time diagnostic tools (Lu et al., 2017). Tensor voting schemes have been used to enhance object recognition in cluttered environments, improving detection accuracy in agricultural settings (Loss et al., 2009). A hybrid model combining generative adversarial networks (GANs) and CNNs demonstrated a defect detection accuracy of 80%, significantly reducing the need for manual labeling (Chou et al., 2019). AI-driven systems have also been developed to classify insects affecting crops, which is critical for effective pest management (Xia et al., 2018). Furthermore, deep semantic segmentation has been used to classify diseases in mangoes, advancing disease control methods in fruit production (Kestur et al., 2019). A deep CNN model developed for crop pest classification showed high accuracy, successfully detecting pests even in complex and cluttered backgrounds (Khan et al., 2024). These advancements showcase how AI and deep learning are revolutionizing crop monitoring by enabling accurate and automated detection systems.

#### 3.5.3 Remote sensing and drone monitoring technologies

Remote sensing and drone technologies have also revolutionized crop monitoring, allowing for large-scale, real-time data collection. UAVs, equipped with remote sensing technologies, have been employed to detect fruits in natural environments, using spatial-frequency analysis and multiview geometry (Rakun et al., 2011). These techniques have also been integrated into smart manufacturing systems for apple classification, enhancing efficiency in fruit production (Ismail et al., 2018). Using remote sensing and machine learning, a model for rice lodging recognition improved prediction accuracy and crop health monitoring (Su et al., 2022). The YOLOv5 model enhanced with a visual attention mechanism achieved high accuracy in recognizing tomato virus diseases, overcoming overfitting and weak generalization (Qi et al., 2022). Additionally, UAVs have been used to detect and manage weeds in crop fields, addressing a major challenge in crop productivity (Das et al., 2022). Furthermore, hyperspectral imaging through UAVs has provided valuable insights into crop health, enabling more targeted interventions for stress levels and nutrient deficiencies (Deng et al., 2022).

#### 3.5.4 Agricultural IoT and automation systems

Agricultural IoT and automation systems represent another significant advancement in crop monitoring, enabling real-time data collection and automated decision-making for efficient crop management. IoT devices have been deployed to monitor various crop parameters, such as soil moisture, temperature, and humidity, facilitating better management practices (Raffik et al., 2024). Robotic systems integrated with IoT sensors have been developed for the automated harvesting of strawberries, enabling more precise and labor-efficient harvesting (Chaivivatrakul and Dailey, 2014). Machine learning algorithms have also been applied to automate silkworm sex discrimination, improving efficiency in sericulture (Guo et al., 2023). A multi-category fruit detection system, utilizing image region proposal networks (RPN), achieved high accuracy and successfully handled overlapping fruits and complex backgrounds (Yu et al., 2019). IoT and drone technologies have been combined in precision farming, offering continuous monitoring and data-driven decision-making for crop health management (Sa et al., 2018). Additionally, real-time irrigation systems based on IoT-based sensors have been developed to optimize water usage while improving crop yields (Abd-Elrahman et al., 2022).

These four subfields—crop image processing and computer vision, deep learning and AI in crop monitoring, remote sensing and drone technologies, and agricultural IoT and automation systems—illustrate how modern technologies are reshaping the landscape of crop recognition and monitoring. These technologies not only enhance the efficiency and accuracy of crop management but also pave the way for more sustainable agricultural practices. Each of these subfields leverages state-of-the-art techniques to address key challenges in agriculture, including pest control, disease management, and yield prediction.

### 3.6 Crop image processing and computer vision

**Figure 13:**
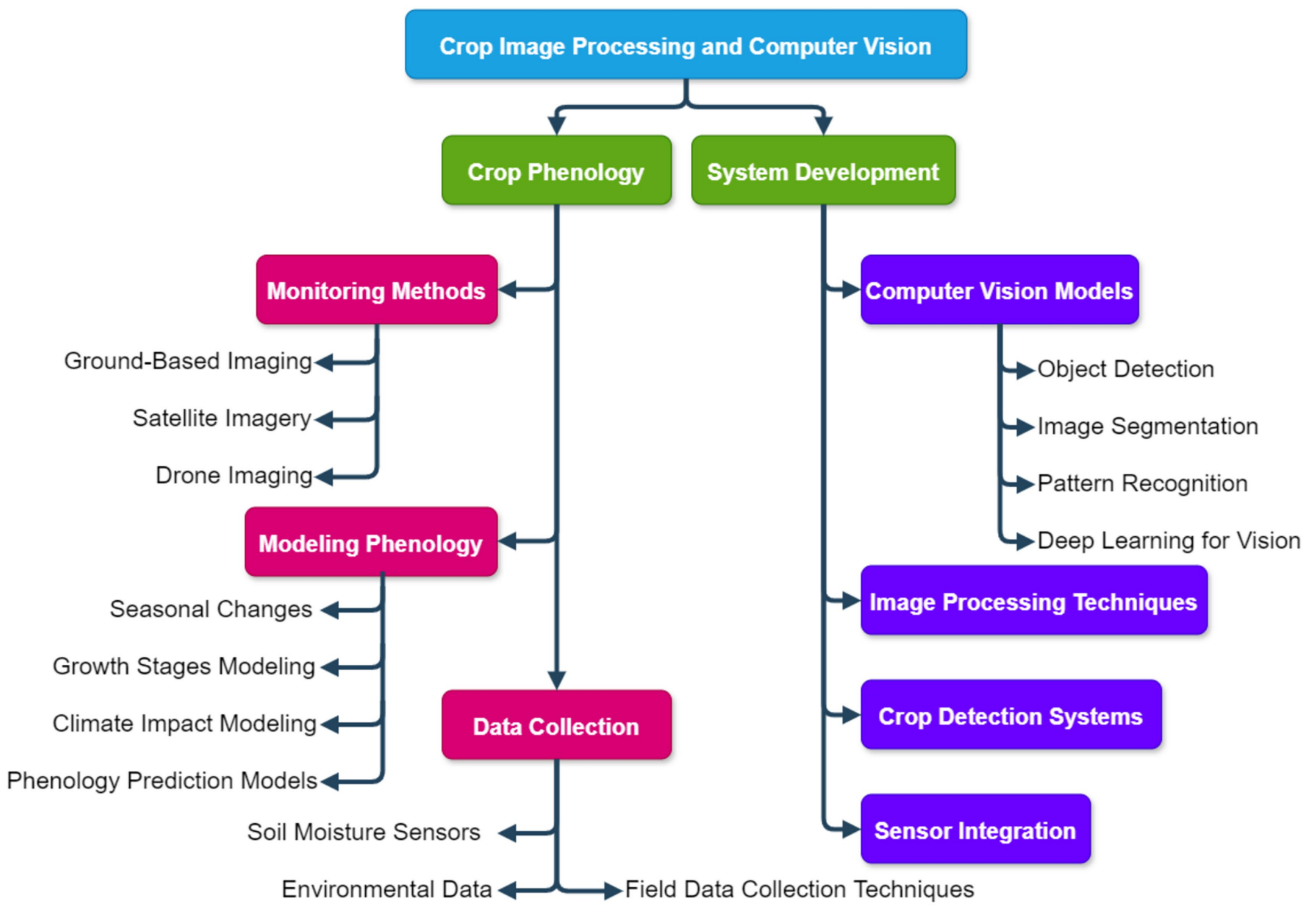
Computational agriculture research mapping: computer vision applications in crop image analysis.

In the field of Crop Image Processing and Computer Vision, research is concentrated around two main directions: Crop Phenology and System Development. These studies leverage image processing technologies, computer vision models, and data collection methods to provide key technological support for precision agriculture and crop monitoring (Figure 13).

Crop Phenology research focuses on monitoring crop growth and development through imaging technologies, including seasonal changes, climate impacts, and other growth factors. Specific areas of research include ground-based imaging, satellite imagery, drone imaging, and remote sensing technologies for crop growth monitoring. For instance, research indicates that ground-based imaging technology can accurately capture the growth status of crops, thus optimizing crop management (Chaw and Mokji, 2017). Moreover, satellite imagery has been applied to monitor the overall growth status of crops, providing crucial data support for agricultural decision-making (Xue et al., 2024). Drone imaging and remote sensing technologies are widely used in crop monitoring, especially for real-time monitoring of large-scale agricultural fields (Abdullahi et al., 2015). In addition, modeling crop phenology has gained significant attention. By constructing climate impact models, crop growth stage models, and seasonal change prediction models, researchers are able to better understand crop growth patterns and predict future growth trends (Guo et al., 2024). Research has also explored the role of climate impact modeling on crop growth cycles, shedding light on the potential effects of climate change on agricultural production (Li et al., 2024). In terms of data collection, crop phenology relies heavily on advanced sensors and data collection technologies, particularly for soil moisture, environmental data, and field data collection. Soil moisture sensors and environmental data collection techniques are key components of the research, enabling precise monitoring of the environmental conditions affecting crop growth (Beck et al., 2021). Field data collection techniques also play a crucial role, providing valuable data for in-depth studies in crop phenology (Jiang et al., 2020).

On the System Development side, the focus is primarily on the development of computer vision models, leveraging deep learning and pattern recognition technologies to enhance crop image processing. For example, object detection and image segmentation techniques are widely used in crop disease detection and crop classification (Liu D. et al., 2025). Furthermore, deep learning has been applied to train image processing models, improving the accuracy of crop monitoring through efficient image recognition techniques (Ahmad et al., 2021). Research on crop detection systems is focused on integrating various image processing technologies and machine learning models to develop automated systems for precision agriculture (Dong et al., 2024). These systems are capable of real-time analysis of crop images, identifying different crop types or diseases. The study of sensor integration addresses the challenge of integrating multiple sensor data into crop monitoring systems to enhance the precision and efficiency of monitoring (Alahi et al., 2023).

In summary, research in Crop Image Processing and Computer Vision has provided crucial support for the advancement of precision agriculture and crop phenology, particularly in crop monitoring, environmental monitoring, data analysis, and system development. These efforts are driving the automation and intelligence of agricultural production processes.

In the field of agricultural image processing and computer vision, scholars have continually explored advanced techniques to improve crop assessment and pest monitoring efficiency. A computer vision-based system was developed for automated evaluation of corn crop quality, combining image processing with machine learning algorithms to assess aspects such as health, size, and ripeness. This system significantly enhances the accuracy and efficiency of crop quality monitoring, although it faces challenges in adapting to varying environmental conditions. To address these challenges, a method for pest detection in winter grains was introduced, utilizing image classification and machine learning algorithms. By strengthening image preprocessing, this approach improved classification accuracy under different lighting and background conditions, demonstrating its robustness in real-world applications (Pérez et al., 2017). In addition, a multimodal produce recognition system was proposed that fuses tactile sensor data with visual imagery. This data fusion technique enhanced classification accuracy, particularly in recognizing produce with complex physical attributes, thus paving the way for more reliable agricultural automation (Chaw and Mokji, 2017). Furthermore, a deep convolutional neural network (CNN) was applied to classify vegetables with high efficiency. The system excelled in handling diverse datasets under varied lighting and backgrounds, showcasing the power of deep learning in agricultural image classification (Zhu et al., 2018). Similarly, a multi-class fruit detection system based on region proposal networks (RPN) was developed, overcoming the limitations of traditional methods by accurately detecting multiple fruit classes even in cluttered and overlapping scenes. This method significantly outperformed prior techniques (Kuang et al., 2018). Finally, a CNN-based crop pest classification system was introduced, capable of accurately identifying and categorizing different pests, even in complex and cluttered environments. This model demonstrated high robustness and accuracy, making it an effective tool for pest monitoring (Thenmozhi and Reddy, 2019). Collectively, these studies illustrate the growing potential of computer vision and deep learning technologies to revolutionize agriculture, enhancing automation and precision in crop and pest management. Table 4 shows several typical cases in the documents.

**Table 4:** Representative applications of computer vision in crop image processing.

References	Purpose	Key technology	Research idea	Conclusion
	Develop a computer vision system for evaluating corn crops.	Computer vision, image processing	The research developed a computer vision system by using cameras to capture images of corn plants under different conditions. The images were processed using machine learning algorithms to detect various aspects of the corn’s health, size, and ripeness. A system was built to analyze the features of the images, providing actionable insights for farmers.	The system successfully evaluated corn crop quality, identifying various health indicators and offering a high level of accuracy in automated assessment of crops.
Pérez et al. (2017)	Classify winter grain pests using image processing.	Image classification, machine learning	The study implemented an image classification system where images of grain crops and pests were collected under different environmental conditions. A machine learning model was trained to recognize pest species from these images. The researchers enhanced the model by using various image pre-processing techniques to ensure that the pest classification was robust and adaptable to diverse scenarios.	The method demonstrated effective pest detection in winter grains, with high classification accuracy, and the model proved to be robust under varying conditions.
Chaw and Mokji (2017)	Analyze a recognition system for produce using tactile and visual data.	Produce recognition, tactile and visual data fusion	The research involved the fusion of tactile data and visual imagery for produce recognition. Tactile sensors were used to gather physical attributes of the produce (e.g., size, texture), while cameras captured visual features. A data fusion approach was employed to merge these two datasets, which were then input into a machine learning model to classify and identify produce with higher accuracy than using either modality alone.	The system improved recognition accuracy by using both tactile and visual cues, offering more precise and reliable results in produce classification compared to single-modal approaches.
Zhu et al. (2018)	Classify vegetables using deep learning methods from images.	Deep learning, image classification	The study applied deep learning techniques, particularly Convolutional Neural Networks (CNN), to classify various types of vegetables based on images. The system was trained on a large dataset of vegetable images under different lighting conditions and backgrounds. The model was fine-tuned to enhance its performance and generalization ability. Additionally, pre-processing steps such as image normalization and augmentation were employed to improve the training process.	Deep learning techniques, especially CNN, led to high-performance classification of vegetables. The model achieved accurate classification even with challenging datasets, such as varied lighting or backgrounds.
Kuang et al. (2018)	Develop a system for multi-class fruit detection using image region proposal networks.	Image region proposal networks, multi-class detection	The research focused on using Region Proposal Networks (RPN) to detect multiple classes of fruits in natural scenes. RPN was combined with Convolutional Neural Networks (CNNs) to generate regions of interest in images, which were then classified into different fruit categories. The system was trained using a diverse dataset of fruit images, and different techniques, such as data augmentation and anchor box optimization, were applied to increase detection performance.	The system efficiently detected multiple fruit classes, achieving high accuracy in real-world conditions. It outperformed traditional methods by effectively dealing with overlapping fruits and varying lighting conditions.
Thenmozhi and Reddy (2019)	Classify crop pests using deep convolutional neural networks (CNN).	Deep CNN, pest classification	The study developed a deep Convolutional Neural Network (CNN) model for classifying crop pests based on images. A large dataset of pest images was collected from different crop types under various environmental conditions. The CNN was trained to extract relevant features from the images and classify pests into different categories. The model’s architecture was optimized to ensure both speed and accuracy, and techniques like dropout and batch normalization were applied to prevent overfitting.	The CNN model effectively classified different crop pests with high accuracy. It was able to recognize pests even in complex and cluttered backgrounds, making it a reliable tool for pest monitoring in agriculture.

### 3.7 Deep learning and artificial intelligence in crop monitoring

**Figure 14:**
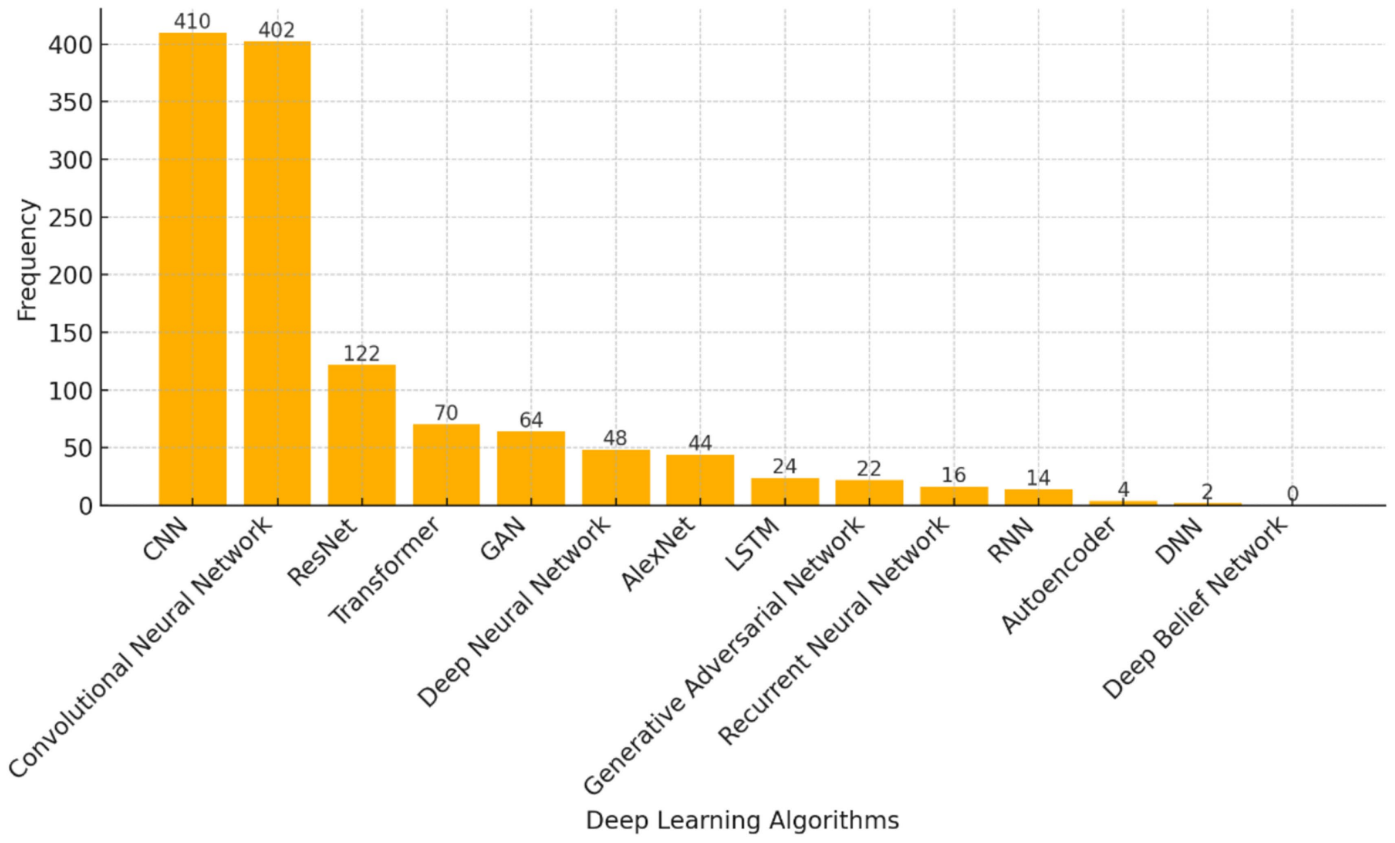
Distribution of deep learning algorithms in agricultural computer vision literature.

The bar chart presents the frequency of various deep learning algorithms used in crop monitoring and agricultural applications, as observed across multiple studies (Figure 14). Convolutional Neural Networks (CNN) and their variants, such as the traditional “Convolutional Neural Network” and “CNN,” were the most frequently employed algorithms, with the highest counts of 402 and 410, respectively. This widespread use of CNN is consistent with findings from various research, where CNNs have been shown to excel in image classification, plant disease detection, and crop segmentation tasks (Rakun et al., 2011; Zhu et al., 2018). Deep Neural Networks (DNN) followed with a count of 48, demonstrating a moderate level of usage for tasks requiring more complex feature extraction and representation learning (Ferreira et al., 2019; Majeed et al., 2020). Recurrent Neural Networks (RNN) were also used but with lower frequency (16 occurrences), indicating their more specialized application in time-series analysis for crop growth prediction (Shi et al., 2019; Hamidisepehr et al., 2020). Generative Adversarial Networks (GANs), Autoencoders, and other more specialized algorithms were less frequently mentioned, suggesting their emerging use in crop monitoring and further exploration in future research (Lu et al., 2015; Wu et al., 2020). The chart highlights the dominance of CNN in the field, reflecting its effectiveness in image-based tasks such as plant health assessment and pest detection, and its continued relevance in advancing agricultural automation through artificial intelligence.

In recent years, deep learning and artificial intelligence have emerged as pivotal tools for enhancing crop monitoring and agricultural automation. One significant challenge is the accurate classification of vegetables, which often varies under different environmental conditions. To address this, a deep learning-based approach using Convolutional Neural Networks (CNNs) was developed, demonstrating high classification performance even in complex lighting and background settings (Zhu et al., 2018). A related challenge involves the segmentation of plant structures for better understanding and analysis. Deep learning-based segmentation models have been applied to separate various plant parts, improving the precision of plant structure analysis and offering more accurate segmentation (Shi et al., 2019). Additionally, plant disease detection, traditionally reliant on labeled data, has been transformed through unsupervised deep learning techniques. These methods detect diseases without the need for labeled training data, enhancing scalability and efficiency in large-scale agricultural settings (Ferreira et al., 2019). Another area where deep learning has proven beneficial is in fruit detection, where deep learning-based segmentation networks have automated the identification and segmentation of fruits, paving the way for more efficient fruit harvesting (Chou et al., 2019). The detection of crop pests, a critical concern in agriculture, has also been improved through object detection methods based on deep learning. These models provide high accuracy, surpassing traditional methods in pest detection (Hamidisepehr et al., 2020). Furthermore, deep learning combined with remote sensing has been utilized to extract valuable apple tree crown information, assisting in precision agriculture by enabling more accurate crop monitoring and management (Wu et al., 2020). Collectively, these studies highlight the transformative potential of deep learning and artificial intelligence in agricultural automation, addressing key challenges in crop classification, disease detection, pest identification, and plant monitoring (Table 5).

**Table 5:** Representative applications of deep learning and AI in crop monitoring.

References	Purpose	Key technology	Research idea	Conclusion
Zhu et al. (2018)	Classify vegetables using deep learning techniques for better performance in varied conditions.	Deep learning, Convolutional Neural Networks (CNN)	Applied CNN to classify vegetables under different lighting and backgrounds. The model was trained on large datasets and optimized for high accuracy.	Deep learning methods showed high classification performance, even under challenging conditions.
Shi et al. (2019)	Develop a segmentation model using deep learning for understanding plant structure.	Deep learning, image segmentation	The model used deep learning for plant-part segmentation, incorporating image processing and neural networks to segment different parts of the plant.	The deep learning-based segmentation model significantly improved plant structure understanding and segmentation accuracy.
Ferreira et al. (2019)	Detect plant diseases automatically using unsupervised deep learning techniques.	Unsupervised learning, Deep Neural Networks (DNN)	Utilized unsupervised DNN models to detect diseases in plants, requiring no labeled training data. This approach aimed to improve scalability and efficiency.	Unsupervised deep learning showed potential for large-scale, automatic disease detection with minimal labeled data.
Chou et al. (2019)	Segment and detect fruits in automated agricultural settings using deep learning.	Deep learning, segmentation networks	Applied deep learning segmentation networks to identify and segment fruits in images, enabling automation in fruit harvesting.	The system successfully detected and segmented fruits, improving automation in agricultural practices.
Hamidisepehr et al. (2020)	Compare different object detection methods for identifying crop pests.	Object detection, Deep learning	Compared several object detection techniques, including deep learning-based models, to classify and detect crop pests from images.	Deep learning-based object detection outperformed traditional methods, providing high accuracy for pest detection.
Wu et al. (2020)	Use remote sensing and deep learning to extract apple tree crown information for crop monitoring.	Deep learning, remote sensing	Combined remote sensing data with deep learning models to extract tree crown information, aiding in precision agriculture.	The approach successfully extracted tree crown features, contributing to better crop monitoring and management.

### 3.8 Remote sensing and drone monitoring technologies

One critical issue in orchard management is the detection of fruit tree pests, which can now be addressed using deep learning algorithms applied to remote sensing data, improving pest detection accuracy and efficiency (Chen et al., 2021). Similarly, rice lodging, a major issue affecting crop yield, has been tackled through the development of machine learning models that leverage remote sensing data, offering a more accurate and scalable solution for monitoring crop health and ensuring better management practices (Su et al., 2022). Moreover, maize tassel detection, vital for crop management, has been significantly improved by utilizing drone imagery combined with remote sensing techniques, allowing for precise identification of maize tassels and supporting better yield prediction (Zhang X. et al., 2023). In orchard management, the use of drone and remote sensing technologies has enabled the efficient mapping of individual fruit trees, facilitating better monitoring and resource management (Chou et al., 2019). Finally, rice seedling distribution, a crucial aspect of crop management, has been predicted with high precision using remote sensing, contributing to improved crop planning and yield optimization (Xiong et al., 2024). Collectively, these studies demonstrate the growing potential of remote sensing and drone monitoring technologies in revolutionizing agricultural practices, providing solutions that enhance both the accuracy and efficiency of crop monitoring and management (Anandakrishnan et al., 2025) (Table 6).

**Table 6:** Representative applications of remote sensing and drone-based monitoring in agriculture.

References	Purpose	Key technology	Research idea	Conclusion
Chen et al. (2021)	Identify fruit tree pests using deep learning and remote sensing data.	Deep learning, remote sensing	Used deep learning algorithms to analyze remote sensing data for detecting fruit tree pests, providing a robust pest identification solution.	Successfully detected fruit tree pests with high accuracy, demonstrating the potential of deep learning in pest management.
Su et al. (2022)	Improve rice lodging recognition with remote sensing data and machine learning.	Remote sensing, machine learning	Developed a model using remote sensing images to recognize rice lodging, improving prediction accuracy and crop health monitoring.	Achieved improved rice lodging recognition, enhancing monitoring and management of crop health.
Zhang X. et al. (2023)	Detect maize tassels using drone-based imagery and remote sensing data.	Drone imagery, remote sensing	Applied drone-based imagery and remote sensing for detecting maize tassels, aiding crop management and improving yield prediction.	Successfully identified maize tassels using drone and remote sensing, aiding crop management.
Chou et al. (2019)	Segment and detect fruits in automated agricultural settings using deep learning.	Deep learning, segmentation networks	Applied deep learning segmentation networks to identify and segment fruits in images, enabling automation in fruit harvesting.	The system successfully detected and segmented fruits, improving automation in agricultural practices.
Xiong et al. (2024)	Map individual fruit trees using drone and remote sensing technologies.	Drone-based remote sensing, spatial analysis	Utilized drone and remote sensing data to map the location and distribution of individual fruit trees, enhancing orchard management.	Mapped individual fruit trees efficiently, improving orchard management and monitoring.
Anandakrishnan et al. (2025)	Precisely predict rice seedling distribution using remote sensing techniques.	Remote sensing, precision agriculture	Leveraged remote sensing to predict the spatial distribution of rice seedlings, improving crop management and precision agriculture.	Accurately predicted rice seedling distribution, contributing to better crop management and planning.

**Figure 15:**
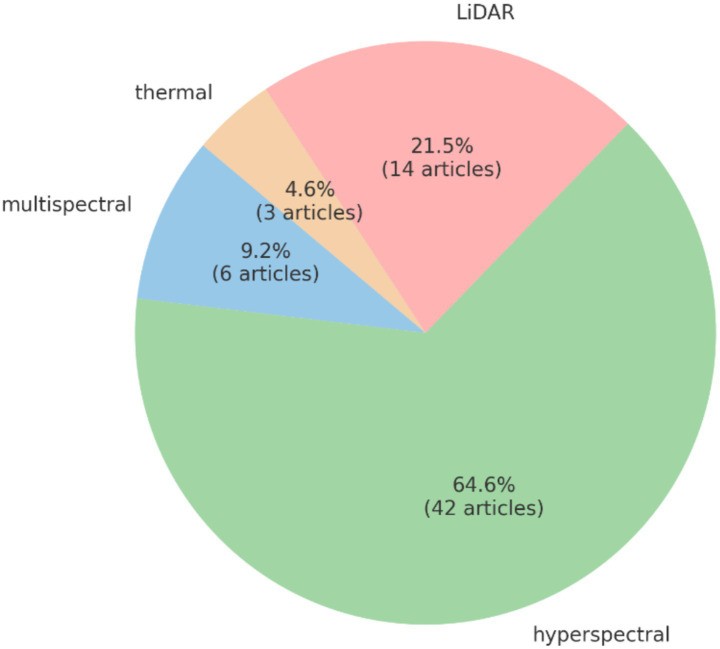
Distribution of remote sensing technologies in agricultural monitoring literature.

The pie chart presents the distribution of remote sensing technologies used in crop monitoring across the literature (Figure 15). Multispectral and hyperspectral technologies dominate, with multispectral sensing being widely applied for vegetation index calculation and pest detection, as shown in several studies (Hao et al., 2025). Hyperspectral sensing, known for its high spectral resolution, is primarily used for material identification and disease detection, contributing to more detailed crop analysis (Meng et al., 2022; Tang et al., 2022; Zhang et al., 2024). While less common, LiDAR technology is gaining traction in applications such as topographic mapping and biomass estimation, with significant potential for precision agriculture (Jayakumari et al., 2021; Wang et al., 2022). Thermal sensing, although mentioned in fewer studies, plays a crucial role in detecting temperature variations, such as water and heat stress in crops (Gill and Khehra, 2020). These technologies collectively offer a comprehensive suite of tools for enhancing crop monitoring, improving decision-making, and fostering precision agriculture practices.

### 3.9 Agricultural IoT and automation systems

**Figure 16:**
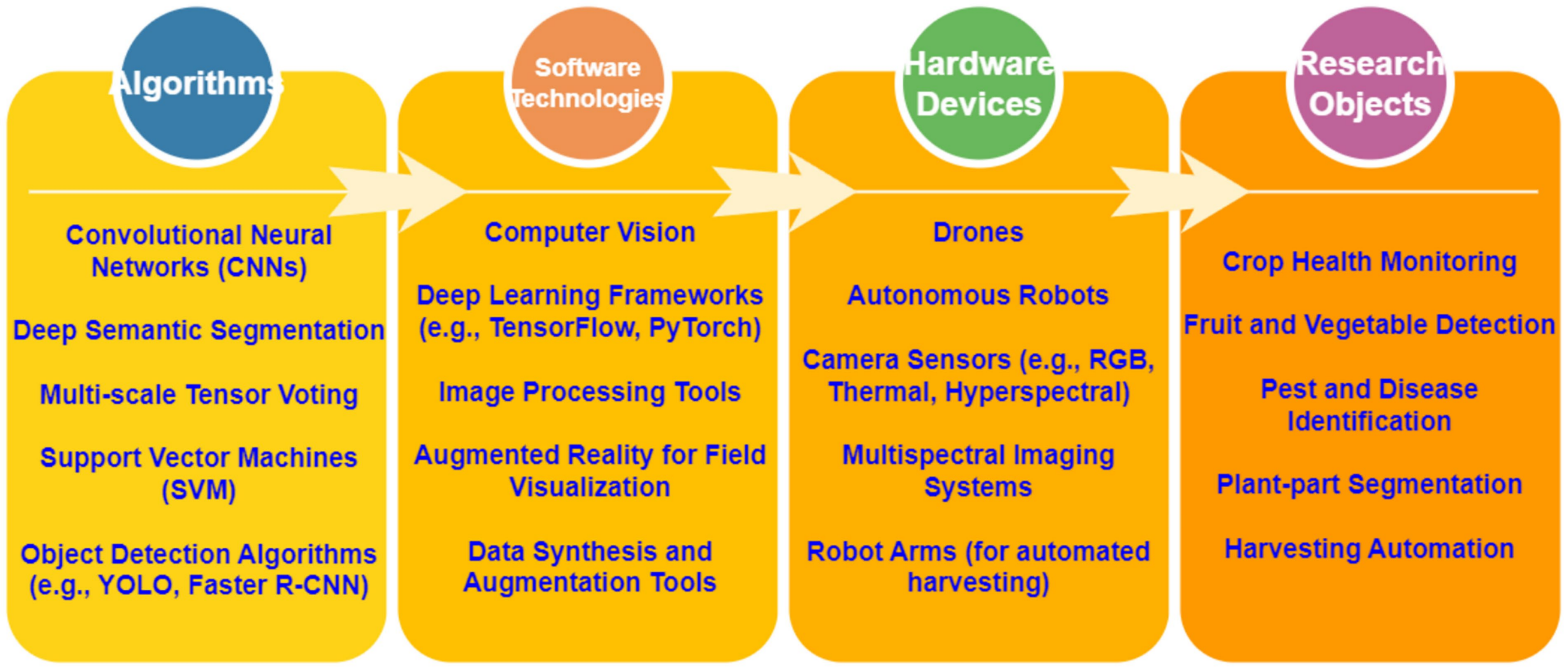
System architecture of agricultural IoT and automation networks.

Based on the technologies identified in the diagram, the integration of algorithms, software technologies, hardware devices, and research objects forms the backbone of modern agricultural IoT and automation systems, driving advancements in precision agriculture (Figure 16). As highlighted in recent studies, the application of Convolutional Neural Networks (CNNs) for image-based analysis is pivotal in automating tasks such as fruit and vegetable detection, pest and disease identification, and crop health monitoring (Loss et al., 2009). These deep learning algorithms, particularly Deep Semantic Segmentation, enable highly accurate pixel-level classification in images captured by drones and autonomous robots, allowing for precise plant-part segmentation (Rakun et al., 2011). In terms of hardware devices, drones have emerged as a critical component in agricultural automation systems, providing mobility and the ability to capture high-resolution data through camera sensors such as RGB, thermal, and hyperspectral imaging (Chaivivatrakul and Dailey, 2014). Coupled with autonomous robots and robot arms for automated harvesting, these systems enhance the efficiency of tasks like fruit picking and pest monitoring, reducing human intervention (Lu et al., 2015). The implementation of multispectral imaging systems further improves the ability to monitor crop health and growth stages, providing a detailed analysis of soil and plant conditions (Zhu et al., 2018). On the software side, computer vision algorithms are employed to process the large amounts of data generated by these devices. Techniques such as data synthesis and augmentation tools improve model training, especially in environments with limited real-world data, ensuring the robustness of detection systems under various conditions (Xiong et al., 2024). Moreover, the integration of augmented reality (AR) into field visualization allows for enhanced interaction with real-time data, assisting farmers in decision-making processes (Loss et al., 2009). Finally, the overall research objects, including crop health monitoring, fruit and vegetable detection, and harvesting automation, rely on these integrated technologies to achieve greater accuracy and efficiency in modern agriculture. As these systems continue to evolve, an enhancement in productivity and sustainability across various agricultural practices is expected, aligning with broader trends toward smart farming (Raffik et al., 2024). The continued development of these technologies will likely lead to a more autonomous, data-driven agricultural ecosystem, where machines and AI systems work in tandem to improve agricultural outputs while minimizing resource use.

Recent advancements in the detection and classification of agricultural products have made significant strides, especially in fruit and bean inspection (Table 7). Researchers have leveraged convolutional neural networks (CNNs) to enhance real-time detection capabilities for various agricultural applications. A novel approach by integrating multi-scale feature fusion and lightweight networks has shown high-precision results in apple segmentation and defect detection (Kang and Chen, 2019). Further optimization was achieved through the combination of generative adversarial networks (GANs) and CNNs, demonstrating an improvement in defect detection accuracy for coffee beans (Chou et al., 2019). In another study, apple classification was enhanced by utilizing spatial pyramid matching (SPM) combined with support vector machines (SVM), achieving a classification accuracy of 98.15% (Ismail et al., 2018). Additional improvements in multi-class fruit detection utilized region-based convolutional neural networks, leading to a miss rate as low as 3.77% (Kuang et al., 2018). Moreover, the design of a shallow CNN structure for apple classification allowed for reduced parameter counts while maintaining competitive accuracy rates (Li et al., 2020). These efforts indicate a promising future for applying deep learning techniques to precision agriculture, improving both efficiency and accuracy in real-time monitoring and inspection systems (Zhang et al., 2020).

**Table 7:** Benchmark comparison of agricultural IoT and automation systems.

References	Purpose	Key technology	Research idea	Conclusion
Kang and Chen (2019)	Realize real-time detection and segmentation of apples and branches in apple orchards for automated picking robots.	Use an improved convolutional neural network (DaSNet-v2) for instance segmentation and semantic segmentation.	Combining multi-scale feature fusion and lightweight network design to optimize detection and segmentation performance.	The system achieved high-precision detection (87.3%) and fast processing (55 ms) in complex environments.
Chou et al. (2019)	Automated detection of coffee bean defects reduces manual labeling costs.	Optimize defect detection models by combining generative adversarial networks (GANs) and data augmentation techniques.	Generate synthetic data through GAN and train deep learning models for defect classification.	The model accuracy reaches 80%, significantly reducing the need for manual annotation.
Ismail et al. (2018)	Develop an apple classification system based on visual sensors for automatic sorting in intelligent manufacturing.	Use Spatial Pyramid Matching (SPM) and Support Vector Machine (SVM) for feature extraction and classification.	Compare multiple image recognition methods and select the optimal classifier.	SPM + SVM classification accuracy reaches 98.15%, suitable for industrial applications.
Kuang et al. (2018)	Improve the accuracy of multi category fruit detection and adapt to complex backgrounds and occlusion conditions.	Generate candidate regions by combining multi feature fusion and improved EdgeBoxes algorithm.	Optimize detection performance through region selection and weighted feature fusion.	The detection miss rate is as low as 3.77%, which is better than traditional sliding window methods.
Li et al. (2020)	Design a lightweight CNN model for Apple classification, suitable for resource constrained IoT devices.	Using shallow CNN structure to reduce parameter count, combined with block voting strategy to address occlusion issues.	Improve the classification performance of small datasets through data augmentation and transfer learning.	The classification accuracy reaches 92%, and the model is lightweight and resistant to overfitting.
Zhang et al. (2020)	Develop a high-throughput corn ear screening system for automated sorting in seed production.	Optimize feature extraction capability by combining VGG-16 and ResNet-50 dual channel CNN.	Design a dedicated image acquisition device and train a dual channel network to achieve efficient classification.	The classification accuracy is 97.23%, suitable for unstructured environments.

## 4 Discussion

### 4.1 Advancements in machine learning for agricultural disease and pest detection

The application of machine learning, particularly deep learning models, has significantly advanced the detection of crop diseases and pests, offering more efficient and accurate methods compared to traditional agricultural monitoring practices. Over recent years, Convolutional Neural Networks (CNNs) have become the go-to tool for image-based recognition tasks in agriculture, such as disease diagnosis, pest detection, and even crop ripeness classification (Bao et al., 2024). These models can analyze vast amounts of visual data captured from various sources, including drones (Ab Rahman et al., 2019), satellites (Jumaat et al., 2018), and ground-level sensors (Loss et al., 2009), enabling the identification of diseases and pests in real-time with high accuracy.

The main advantage of machine learning techniques in this context is their ability to handle complex and high-dimensional data, enabling the detection of subtle patterns that human experts might miss. For example, deep learning models trained on large datasets of crop images can identify specific symptoms of diseases or pest infestations even under challenging field conditions, such as varying lighting or occlusions. This capability is particularly important for tasks like early disease detection, where timely intervention can prevent significant crop loss. Moreover, these models can be continually improved and adapted to new diseases and pest species, ensuring their long-term utility in agriculture.

However, there are still challenges in deploying these systems on a wide scale. One issue is the variability of environmental conditions, which can affect the performance of machine learning models trained on specific datasets. For instance, models that perform well in one geographical region may struggle when applied to crops grown in different climates or under different soil conditions. In addition, data quality remains a concern; high-quality annotated datasets are essential for training robust models, but such datasets are often limited in agricultural contexts. To address these limitations, recent research has focused on improving model generalization and developing strategies for training models with fewer labeled examples, such as transfer learning and semi-supervised learning. These efforts are crucial for expanding the applicability of AI-based crop monitoring systems across different farming environments, pushing the boundaries of what is possible in agricultural disease and pest management.

### 4.2 Integration of remote sensing technologies with AI for precision agriculture

The integration of remote sensing technologies with artificial intelligence (AI) represents a transformative shift in the monitoring and management of agricultural systems (Alahi et al., 2023). Remote sensing tools, including unmanned aerial vehicles (UAVs) (Deng et al., 2022), satellites (Jumaat et al., 2018), and various sensor technologies (Kang and Chen, 2019), provide high-resolution (Jumaat et al., 2018), multispectral data that (Hao et al., 2025), when combined with AI, can offer unprecedented insights into crop health and environmental conditions (De Angelis et al., 2023). The ability to capture detailed images and sensor data from large-scale agricultural fields allows for continuous, real-time monitoring of crops, which was previously not possible with traditional manual methods (Guo et al., 2023).

Machine learning algorithms, especially deep learning models, are able to process and analyze the vast amounts of data generated by remote sensing platforms. For example, deep convolutional networks can classify crop types, detect diseases, monitor growth stages, and even assess soil conditions from images captured by UAVs. This fusion of AI and remote sensing technology significantly enhances the accuracy and efficiency of crop management practices, enabling farmers to make data-driven decisions that improve yields and reduce resource waste. Furthermore, AI-powered systems can detect patterns and correlations in the data that would otherwise be difficult to uncover, such as subtle changes in crop conditions that might indicate the onset of disease or pest infestation.

Despite the significant potential of these integrated systems, challenges remain. One of the key issues is the complexity of managing and processing the massive datasets generated by remote sensing technologies. These datasets require sophisticated data storage solutions, fast processing power, and highly advanced algorithms for timely analysis. Additionally, the environmental variability between regions—such as differences in climate, topography, and soil conditions—means that AI models often need to be retrained or fine-tuned to perform well in different settings. To address these issues, research is moving toward creating more adaptable and scalable systems that can be customized for different types of crops and farming conditions. Additionally, there is a growing need for models that can handle multi-source data, integrating information from satellite imagery, UAVs, soil sensors, and weather data to provide a more holistic view of crop health and field conditions.

### 4.3 Challenges and future directions in AI-driven agricultural systems

While AI has the potential to revolutionize agriculture, its widespread adoption faces several significant challenges (Abdar et al., 2021a). A critical hurdle is the need for large, high-quality datasets to train machine learning models (Zhang et al., 2020). In agriculture, collecting sufficient labeled data can be particularly challenging due to the diverse and dynamic nature of farming environments (Xiong et al., 2024). Unlike other industries, where datasets can be standardized, agricultural data varies greatly depending on the crop type (Abdali et al., 2024), climate (Abbass et al., 2022), geographical location (Comber et al., 2023), and even specific farm practices (Sa et al., 2018). Furthermore, manual annotation of agricultural data—such as labeling images of crops, diseases, or pests—can be labor-intensive and expensive (Ferreira et al., 2019), making it difficult to create comprehensive datasets that capture all the potential variations in agricultural scenarios.

One challenge is the adaptability and scalability of AI models, particularly in the context of climate change. Models trained on specific conditions may struggle when applied to diverse environments. Barriers such as limited infrastructure and policy support in developing countries hinder technology deployment. However, government incentives and public-private partnerships can accelerate adoption, and current research focuses on developing adaptable models that require less retraining across various conditions. Another challenge lies in the adaptability and scalability of AI models. While deep learning models have demonstrated impressive performance in controlled conditions, their deployment in real-world agricultural environments can be less straightforward. Machine learning models trained on specific crops or conditions may struggle when applied to different environments, leading to issues with model generalization. This is particularly relevant in the context of climate change, which is altering growing conditions across the globe. Developing models that can adapt to a wide range of environmental conditions—without requiring extensive retraining—is a major focus of current research.

Looking to the future, the combination of AI with other emerging technologies, such as robotics and the Internet of Things (IoT), offers exciting possibilities for creating fully autonomous, data-driven agricultural systems. AI-powered robots, for example, could automate tasks like planting, harvesting, and pest control, while IoT devices could monitor environmental factors such as soil moisture, temperature, and nutrient levels in real-time. The integration of these technologies would allow for the creation of smart farming systems that can make real-time decisions based on data, significantly improving efficiency, reducing waste, and optimizing resource usage. However, to fully realize this vision, further advances in hardware, data processing, and system integration are necessary. AI systems must become more robust, scalable, and adaptable to handle the complexities of agricultural environments and meet the diverse needs of farmers worldwide.

In conclusion, while AI and machine learning technologies hold immense potential for transforming agricultural practices, addressing the challenges of data quality, model adaptability, and system integration will be key to their widespread adoption. As research continues to evolve, the convergence of AI, remote sensing, robotics, and IoT will likely usher in a new era of precision agriculture that improves both the productivity and sustainability of farming operations on a global scale.

## 5 Conclusion

This review examines the intersection of machine learning, particularly deep learning, and agricultural information systems, focusing on crop monitoring, disease detection, and pest management. The findings highlight a paradigm shift in agriculture, where deep learning algorithms and remote sensing technologies are redefining agricultural practices. AI models, especially CNNs, are instrumental in automating tasks such as crop health assessment and pest classification, with the potential to enhance sustainability and productivity.

Despite advancements, challenges remain, such as data heterogeneity, model generalization, and limited high-quality datasets. Overcoming these barriers requires strategies like data augmentation, transfer learning, and multi-source data integration. Our experimental results show that AI can significantly improve agricultural applications, but real-world deployment requires further advancements in real-time processing and scalability. Techniques like model compression and Explainable AI (XAI) are essential for effective integration. This study underscores the transformative potential of AI, emphasizing the need for interdisciplinary approaches to address both technical and practical barriers in agriculture. Future research should focus on ensuring that AI-driven solutions are sustainable, equitable, and capable of addressing global food security challenges.
